# Splintless approach improves surgical accuracy in Le Fort I osteotomies: a systematic review and meta-analysis

**DOI:** 10.3389/froh.2026.1854976

**Published:** 2026-06-16

**Authors:** Darius Sandu, Bianca G. N. Cavalcante, Gábor Varga, Bence Szabo, Alexander Schulze Wenning, Mojtaba Dahmardeh, Eniko V. Szabó, Peter Hegyi, Gábor Gerber, Victor-Vlad Costan

**Affiliations:** 1 Centre for Translational Medicine, Semmelweis University, Budapest, Hungary; 2 Department of Surgery, Oral and Maxillofacial Surgery, Faculty of Dental Medicine, “Grigore T. Popa” University of Medicine and Pharmacy, Iași, Romania; 3 Department of Oral Biology, Semmelweis University, Budapest, Hungary; 4 Department of Restorative Dentistry and Endodontics, Semmelweis University, Budapest, Hungary; 5 Institute for Translational Medicine, Medical School, University of Pécs, Pécs, Hungary; 6 Institute of Pancreatic Diseases, Semmelweis University, Budapest, Hungary; 7 Oral Morphology Group, Department of Anatomy, Histology and Embryology, Semmelweis University, Budapest, Hungary

**Keywords:** accuracy, BSSO, CAD/CAM splints, LeFort I, orthognathic surgery, patient-specific implants, surgical templates

## Abstract

**Background:**

Accurate bone repositioning is crucial during orthognathic surgery (OS), as even slight deviations from the planned position can impact function and esthetics. As splintless techniques are rapidly evolving, patient-specific implants (PSI) and digital surgical templates (DST) are establishing new standards. This systematic review and meta-analysis aimed to assess the effectiveness of splintless OS compared to virtually planned splints.

**Materials and methods:**

We conducted our study in accordance with the guidelines of the PRISMA 2020 framework. A systematic search was performed on November 15, 2024, across PubMed, Embase, and Cochrane. We included randomized controlled clinical trials and observational studies. The primary outcome was surgical accuracy, assessed by the mean linear and angular deviation across the three spatial axes.

**Results:**

Among the 8,563 identified studies, sixteen met inclusion criteria, and nine were subsequently pooled for meta-analysis. Findings were statistically significant for splintless OS concerning linear deviation along the anteroposterior (MD = −0.49 mm; 95% CI: [−0.73, −0.26]), vertical (MD = −0.32; 95% CI: [−0.53, −0.11]), and mediolateral (MD = −0.37; 95% CI: [−0.59, −0.15]) axes. In terms of angular deviation, PSI showed significant results with reduced discrepancies around the anteroposterior axis (roll) (MD = −0.48; 95% CI: [−0.88, −0.08]). The results did not achieve statistical significance in yaw (MD = −0.71; 95% CI: [−1.50; 0.09]) or pitch (MD = −0.57; 95% CI: [−1.45; 0.31]). However, surgical accuracy has shown consistent improvement across studies.

**Conclusion:**

Splintless OS consistently showed significantly lower mean deviations from the planned position, outperforming splint-based OS. These results indicate that PSI and DST may offer superior surgical accuracy compared to virtually planned splints. DST may be a valuable, potentially lower-cost alternative, providing the same clinical benefits as PSI.

**Systematic Review Registration:**

https://www.crd.york.ac.uk/PROSPERO/view/CRD42024605292.

## 1 Introduction

Orthognathic surgery (OS) is the surgical repositioning of the jaws to correct skeletal deformities and improve function and facial aesthetics. Accurate positioning of the maxillary bones is paramount, as minor deviations can affect both function and esthetics. The emergence of computer-aided design (CAD) and computer-aided manufacturing (CAM) technologies has revolutionized various fields, including healthcare (1). Three-dimensional virtual surgical planning (3D VSP) has enabled the replacement of traditional intermediate splints with CAD/CAM splints, allowing extensive preoperative simulation (2–4).

Although CAD/CAM splints are intended to improve accuracy, translating the virtual plan into surgery remains challenging and often result in clinically unacceptable deviations of more than 2 mm (4–8). These deviations occur primarily because maxillary repositioning relies on the mandibular bone, the position of the condyles in the glenoid fossa, the need for intraoperative measurements for the vertical positioning of the maxilla, and errors in seating the splint (6, 9).

To overcome all these disadvantages, several splintless approaches have been developed using 3D-VSP and CAD/CAM technologies, including digital surgical templates (DST) (10, 11), patient-specific implants (PSI) (12–14), intraoperative navigation (15), augmented reality/mixed reality (AR/MR) (16, 17). The PSI kit includes a CAD/CAM cutting guide and a customized osteosynthesis plate. The cutting guide indicates the exact planned osteotomy line and the correct placement of the screw holes, while the customized osteosynthesis plate accurately positions the bony segment in all spatial axes (13, 18–20). Alternatively, the DST kit comprises CAD/CAM cutting and repositioning guides. After osteotomy, the repositioning guides are placed on the bone surface, and osteosynthesis is performed with stock plates (21–23). Both bone- and tooth-supported guides have been created (24).

While AR/MR-based workflows showed promising results for improving spatial orientation and reducing operative time in OS, current evidence is limited to early feasibility studies and small cohort studies (25).

Maximizing the benefits of 3D-VSP in OS requires the most accurate transfer of virtual planning to the surgical procedure. Given the discrepancies in the literature and clinical practice, we aimed to evaluate the effectiveness of splintless OS, aided by PSI or DST, compared with virtually planned splints for Le Fort I (LFI) osteotomies and bilateral sagittal split osteotomies (BSSO). This study investigated whether using PSI or DST improves accuracy in these surgical procedures.

## 2 Material and methods

This systematic review and meta-analysis was conducted based on the recommendations of the Cochrane Handbook for Systematic Reviews of Interventions (version 6.5) (26). This study is reported according to the Preferred Reporting Items for Systematic Reviews and Meta-Analysis (PRISMA) 2020 guideline (27), which can be found in the Supplemental Digital Content (SDC) (SDC, Methods 1). We assessed the methodological quality of the review using the AMSTAR 2 checklist (SDC, Methods 2) (28). The review protocol was registered at the International Prospective Register of Systematic Reviews (PROSPERO) (CRD42024605292), and we fully adhered to it.

### 2.1 Eligibility criteria

Studies involving patients undergoing OS via LFI osteotomy, with or without BSSO, using splintless techniques with either PSI (I1) or DST (I2), and compared to virtually planned splints (C), were included. The primary outcome was surgical accuracy (linear or angular deviation from the virtual plan), assessed by superimposing the post-operative computed tomography scan onto the virtual plan. Measurements were conducted at specific dental landmarks, listed in the table of baseline characteristics (SDC, Table S1). Secondary outcomes included operative time, complications, stability, cost-effectiveness, quality of life, patient and surgeon satisfaction, and blood loss. However, only one eligible study reporting surgical accuracy for BSSO was identified during screening (29). As a result, the quantitative synthesis was restricted to LFI osteotomies. Data for the BSSO study were reported narratively where applicable.

Eligible studies included randomized controlled trials (RCTs), non-RCTs, and observational studies that reported primary data with at least one intervention, one control group, and at least one relevant outcome. We excluded studies reporting segmental LFI, syndromic cases, reoperations, children under 16 years of age, case reports, case series, reviews, and abstracts. No filters were applied based on language, publication date, or study quality.

### 2.2 Information sources

Our systematic search was conducted on November 15, 2024, in MEDLINE (via PubMed), Cochrane Library (CENTRAL), and Embase. Neither filters nor restrictions were employed. References of included studies were also searched for eligible studies. The detailed search strategy is available in SDC Methods 3.

### 2.3 Selection process

We screened the publications using EndNote X21 and Clarivate. After automatic and manual duplicate removal, titles, abstracts, and full texts were screened against predefined criteria. Two independent reviewers (DS and MD) conducted the selection, with Cohen's kappa coefficients of 0.90 (title-abstract) and 0.80 (full-text) (30). Disagreements were resolved by a third reviewer (AS).

### 2.4 Data collection process

Data were collected independently from eligible articles by two authors (DS and MD) using a standardized data collection form in an Excel spreadsheet (Office 365; Microsoft, Redmond, WA, USA). We extracted the first author, publication year, country, study design, study population demographic data, planning software, follow-up time, and results for all reported outcomes of interest. Disagreements were resolved by discussion. If data were insufficient, the corresponding authors were contacted.

### 2.5 Study risk of bias assessment and certainty of evidence

The risk of bias was assessed using the Revised Cochrane Risk of Bias Tool for RCTs (RoB 2.0) (31) and the Risk of Bias in Non-randomized Studies of Interventions (ROBINS-I) tool (32) by two independent authors (DS and MD). Disagreements were resolved by discussion. For RoB 2.0, assessments were made across the following domains: D1: Risk arising from the randomization process; D2: Bias due to deviations from intended intervention; D3: Bias due to missing outcome data; D4: Bias in the measurement of the outcome; and D5: Bias in the selection of the reported results. For ROBINS-I, the domains evaluated were: D1: Bias due to confounding; D2: Bias due to selection of participants; D3: Bias in classification of interventions; D4: Bias due to deviations from intended interventions; D5: Bias due to missing data; D6: Bias in measurement of outcomes; and D7: Bias in the selection of reported results.

Plots of the domain-level judgment were created by using robvis tool (33). Certainty of evidence was rated using the Grades of Recommendation, Assessment, Development, and Evaluation (GRADE) methodology (34), with a summary table created in the GRADEPro tool (see SDC, Methods 4) (35).

### 2.6 Synthesis methods

Assuming substantial heterogeneity, we used a random-effects model to pool effect sizes.

For continuous outcomes, mean differences (MDs) with 95% confidence intervals (CIs) were calculated using sample sizes, means, and standard deviations (SDs) (or standard errors, variances, or CIs) extracted separately for each group. When only medians, quartiles, and/or interquartile ranges (IQRs) were reported and contacting the author was unsuccessful, means and SDs were approximated using methods by Luo et al. and Shi et al. (36, 37) All approximations were performed according to standardized methods and reported transparently. For MD calculations, the control group mean values were subtracted from the experimental group mean values. Meta-analyses were performed using studies with comparable three-dimensional measurement methodologies, including superimposition of planned and postoperative CBCT images and assessment of translational and rotational deviations using well-specified anatomical landmarks.

An inverse variance weighting method was used to calculate the pooled MD. Heterogeneity variance measure (*τ*^2^) was estimated using the restricted maximum-likelihood estimator, with the Q-profile method for confidence intervals (38, 39).

The t-distribution-based method was used to calculate CIs for the MDs in each study. Results were summarized in forest plots, and prediction intervals (the expected range of effects of future studies) were reported when appropriate (i.e., when the number of studies was sufficiently large and not too heterogeneous). Statistical significance was assumed if the pooled CIs excluded the null effect.

Additionally, between-study heterogeneity was assessed using Higgins-Thompson's *I*^2^ statistic (40). Funnel plots and Egger's test (*p* < 0.10) assessed small study bias (41). Potential outlier publications were explored using the influence analysis recommended by Harrer et al. (38).

All statistical analyses were performed with R (R Core Team 2021, v4.4.1) (42), using the meta package by Balduzzi et al., 2019 (v8.0-2) (43) for basic meta-analysis calculations and plots, and the dmetar package by Harrer et al., 2019 (v0.1.0) (44) for additional influential analysis calculations and plots.

A subgroup analysis compared PSI and DST vs. splints, using Cochrane's *Q* test (omnibus test) for between-group differences (*p* < 0.05) (38).

## 3 Results

### 3.1 Search and selection

**Figure 1:**
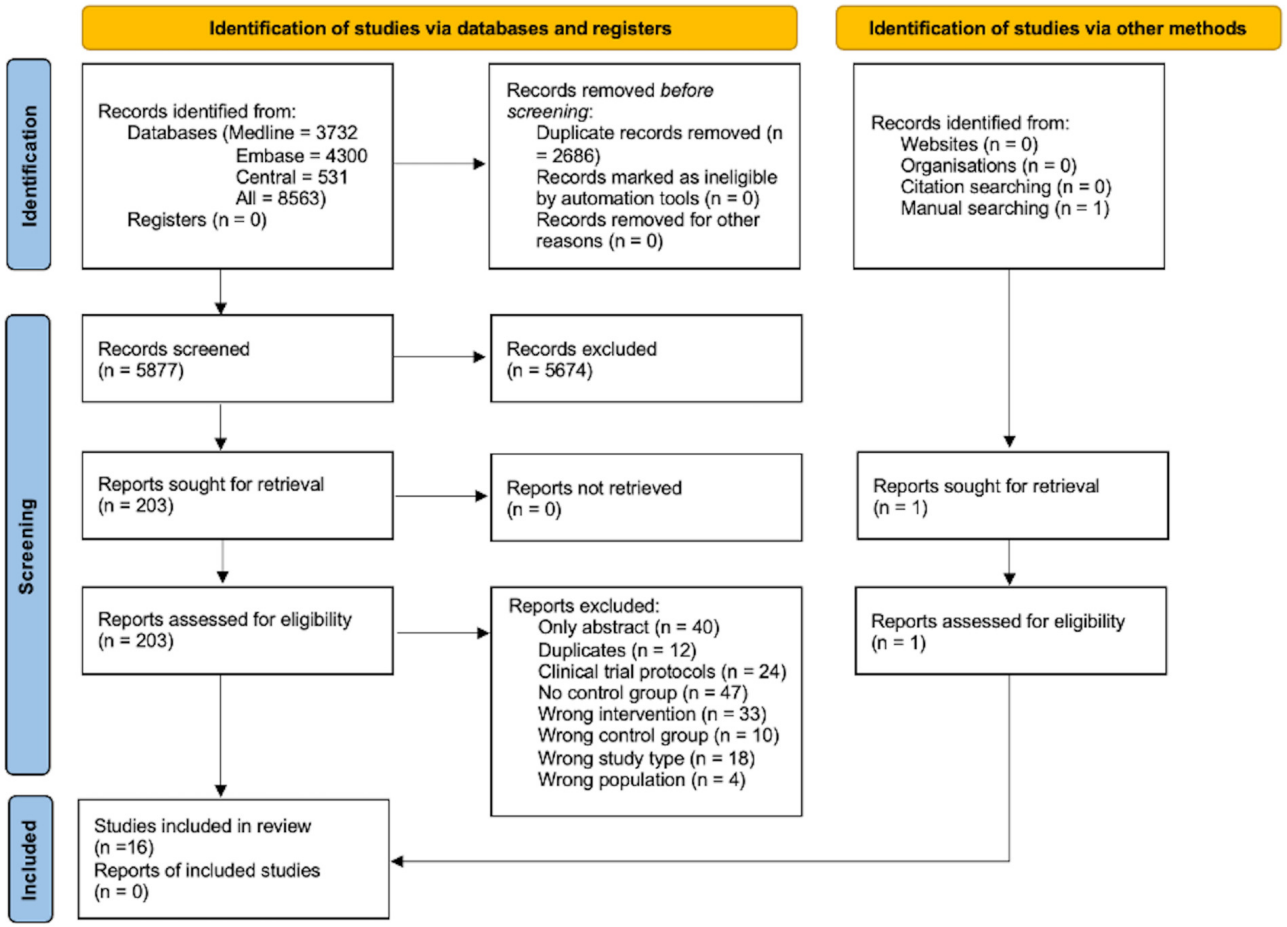
Prisma flowchart of the selection process.

A total of 8,563 studies were identified and screened, and 203 full texts were assessed for eligibility. Sixteen publications met the inclusion criteria, of which nine studies were included in the meta-analysis. During screening, only one study involving BSSO met the inclusion criteria. As a result, no quantitative analysis could be performed for BSSO. Most exclusions were due to unsuitable study design, interventions, populations, or the absence of a control group. The Prisma flowchart details the selection process (Figure 1). In total, 803 patients participated in this study, of which 450 were included in the quantitative analysis and 353 in the systematic review: 389 underwent splintless OS using PSI or DST, while 414 underwent splint-based OS.

### 3.2 Baseline characteristics of included studies

Baseline characteristics of included studies are presented in SDC, Tables S1, S2. Four of the nine studies included in the quantitative analysis were RCTs (45–48), and five were observational (49–53). Seven studies assessed the accuracy of PSI vs. splint-based techniques in non-segmental LFI osteotomies, while two evaluated DST vs. splints.

### 3.3 Primary outcomes

#### 3.3.1 Linear accuracy of non-segmental Le Fort I osteotomies

Linear accuracy was assessed along the *y*- (antero-posterior), *z*- (supero-inferior), and *x*- (medio-lateral) axes. A pooled analysis of 9 studies (450 patients) compared the splintless OS to the splint-based technique. Negative MD values indicate that splintless LFI osteotomies tend to deviate less from the planned position than splint-based surgeries. The certainty of evidence was moderate in the *y*-axis, low in the *x*-axis, and very low in the *z*-axis. (SDC, Methods 4).

**Figure 2:**
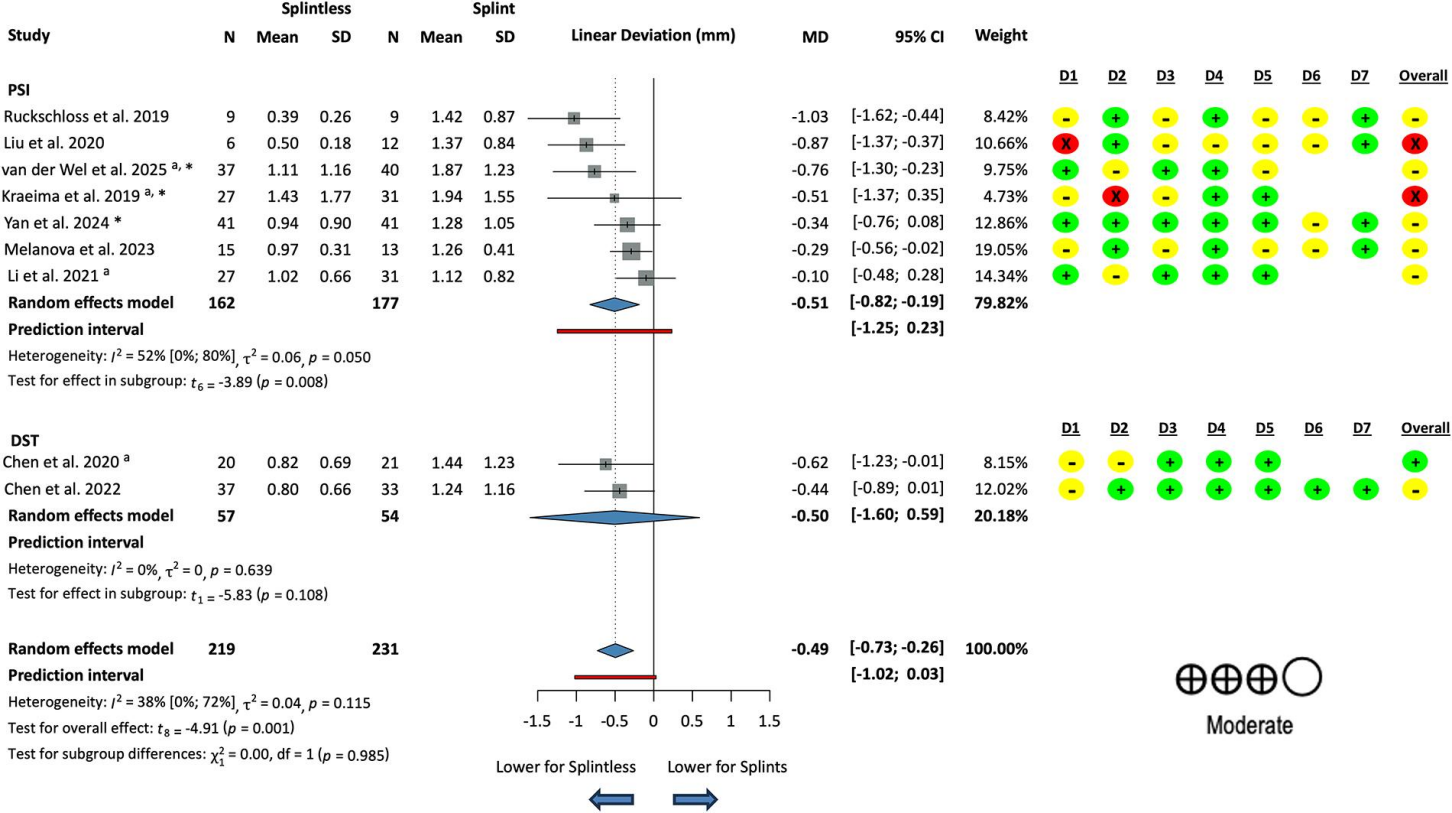
Forest plot demonstrating the effect of splintless (PSI/DST) vs. splint-based approaches in LFI osteotomies on the mean deviation from the planned position (in mm) along the anteroposterior axis. *N*, number of patients in each group; MD, mean difference; SD, standard deviation; CI, confidence interval; PSI, patient-specific implants; DST, digital surgical templates. Studies marked with “*” indicate that means and/or standard deviations were estimated from medians, quartiles, or ranges. Studies marked with “a” are RCTs. Certainty of evidence: moderate.

Along the anteroposterior axis, a statistically significant difference favored splintless OS (MD = −0.49 mm; 95% CI: [−0.73, −0.26]). Subgroup analysis showed that PSI significantly achieved higher accuracy (MD = −0.51 mm; 95% CI: [−0.82, −0.19]), while DST showed a non-significant result (MD = −0.50 mm; 95% CI: [−1.60, 0.59]) due to limited data. This indicates that the accuracy of surgery along the *y*-axis has an overall improvement of 0.51 mm with PSI and 0.5 mm with DST (Figure 2).

**Figure 3:**
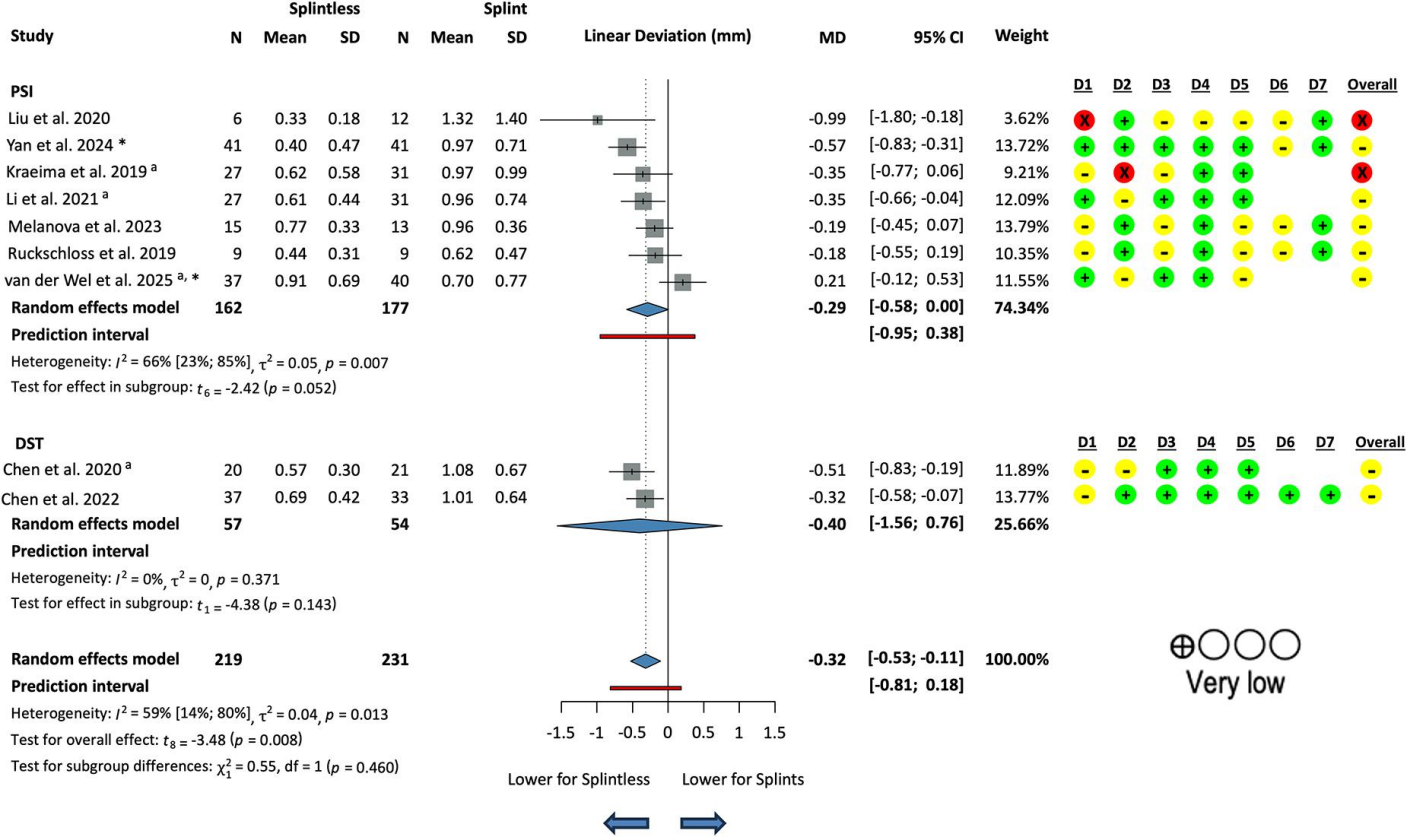
Forest plot demonstrating the effect of splintless (PSI/DST) vs. splint-based approaches in LFI osteotomies on the mean deviation from the planned position (in mm) along the vertical axis. *N*, number of patients in each group; MD, mean difference; SD, standard deviation; CI, confidence interval; PSI, patient-specific implants; DST, digital surgical templates. Studies marked with “*” indicate that means and/or standard deviations were estimated from medians, quartiles, or ranges. Studies marked with “a” are RCTs. Certainty of evidence: very low.

A similar pattern was observed in the vertical axis, where splintless OS significantly improved accuracy (MD = −0.32 mm; 95% CI: [−0.53, −0.11]). PSI maintained significant results (MD = −0.29 mm; 95% CI: [−0.58, 0.00]), while DST did not achieve statistical significance (MD = −0.40 mm; 95% CI: [−1.56, 0.76]) (Figure 3).

**Figure 4:**
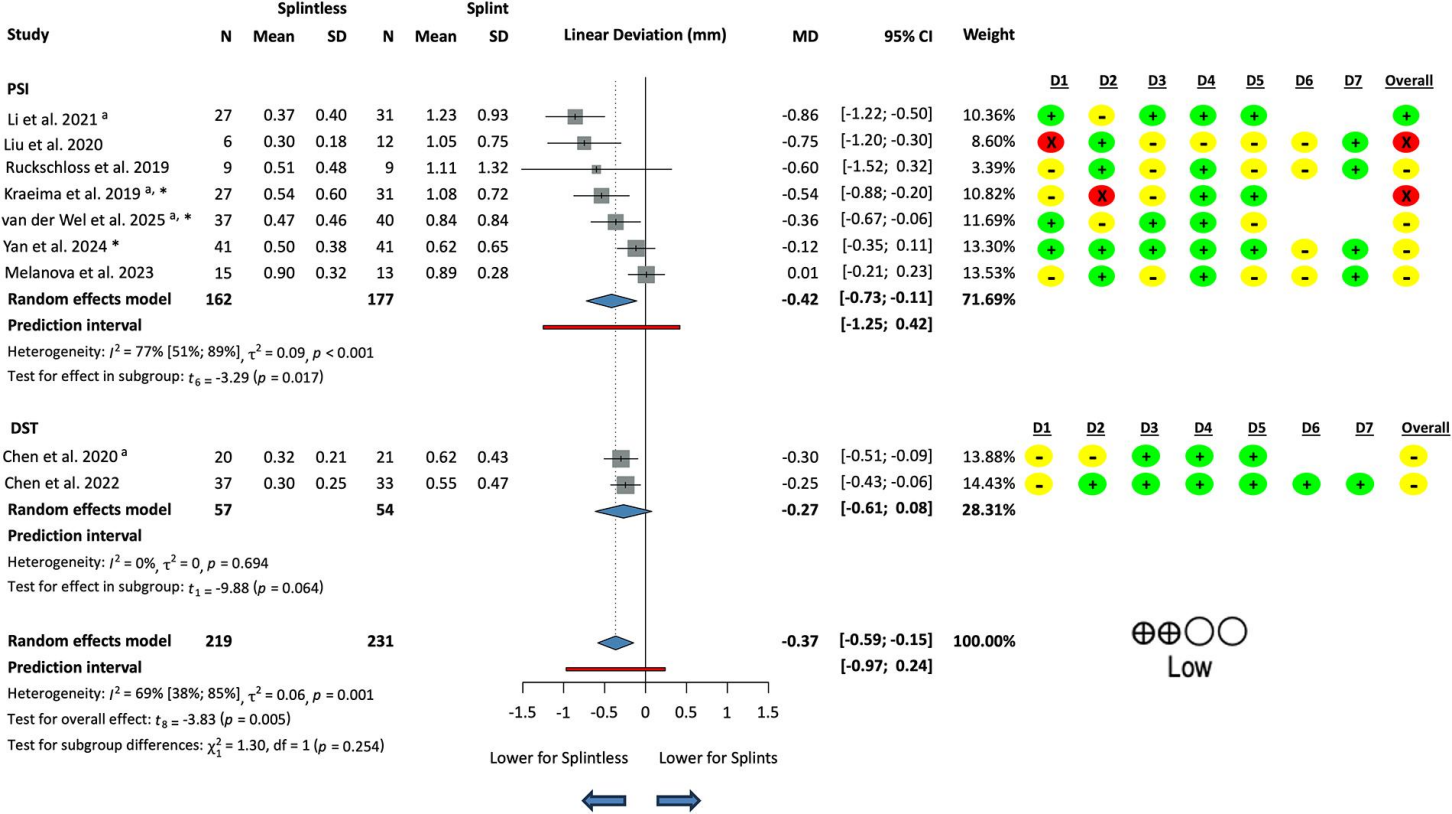
Forest plot demonstrating the effect of splintless (PSI/DST) vs. splint-based approaches in LFI osteotomies on the mean deviation from the planned position (in mm) along the mediolateral axis. *N*, number of patients in each group; MD, mean difference; SD, standard deviation; CI, confidence interval; PSI, patient-specific implants; DST, digital surgical templates. Studies marked with “*” indicate that means and/or standard deviations were estimated from medians, quartiles, or ranges. Studies marked with “a” are RCTs. Certainty of evidence: low.

We observed a statistically significant difference for splintless OS in the mediolateral axis (MD = −0.37 mm; 95% CI: [−0.59, −0.15]). PSI once again yielded a significant result (MD = −0.42 mm; 95% CI: [−0.73, −0.11]), while DST did not (MD = −0.27 mm; 95% CI: [−0.61, 0.08]) (Figure 4).

#### 3.3.2 Angular accuracy in non-segmental Le Fort I osteotomies

Angular accuracy was assessed based on rotational movements around the three axes of space: rotation around the *y*-axis (roll), around the *z*-axis (yaw), and around the *x*-axis (pitch). A pooled analysis of 7 studies involving 339 patients was performed in this case, and we were unable to perform a subgroup analysis for DST as this outcome was not reported. We rated the certainty of evidence as low for roll and very low for pitch and yaw (SDC, Methods 4).

**Figure 5:**
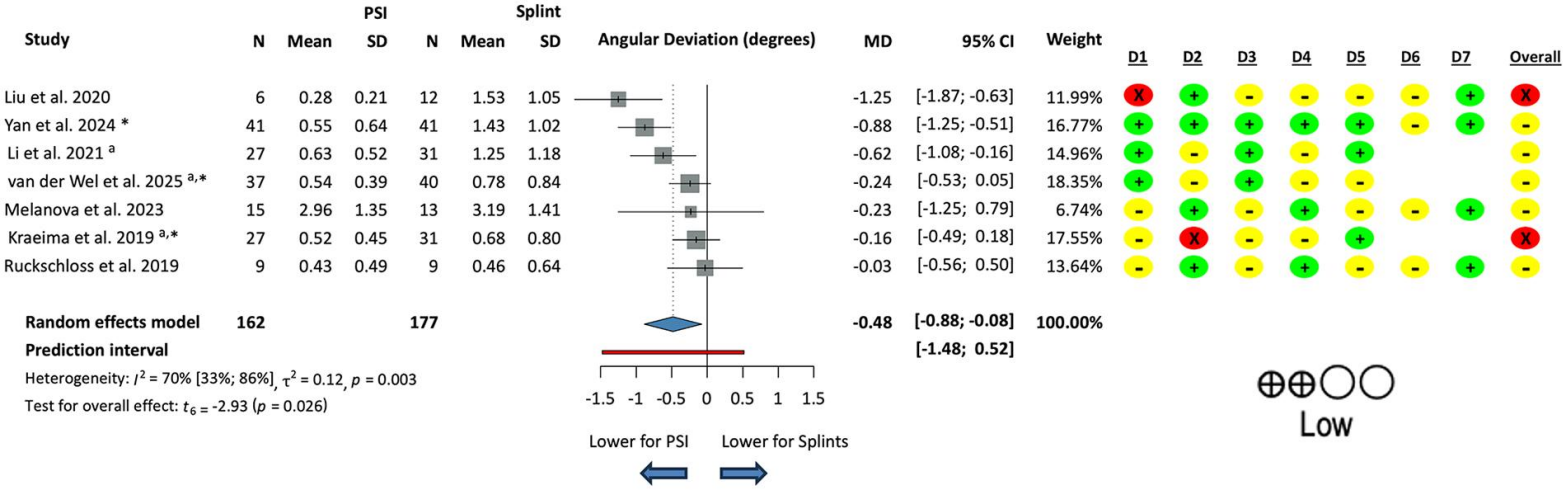
Forest plot demonstrating the effect of PSI vs. splint-based approaches in LFI osteotomies on the mean deviation from the planned position (in degrees) in roll. *N*, number of patients in each group; MD, mean difference; SD, standard deviation; CI, confidence interval; PSI, patient-specific implants. Studies marked with “*” indicate that means and/or standard deviations were estimated from medians, quartiles, or ranges. Studies marked with “a” are RCTs. Certainty of evidence: low.

The accuracy was significantly increased in roll with PSI (MD = −0.48°; 95% CI [−0.88; −0.08]) (Figure 5).

**Figure 6:**
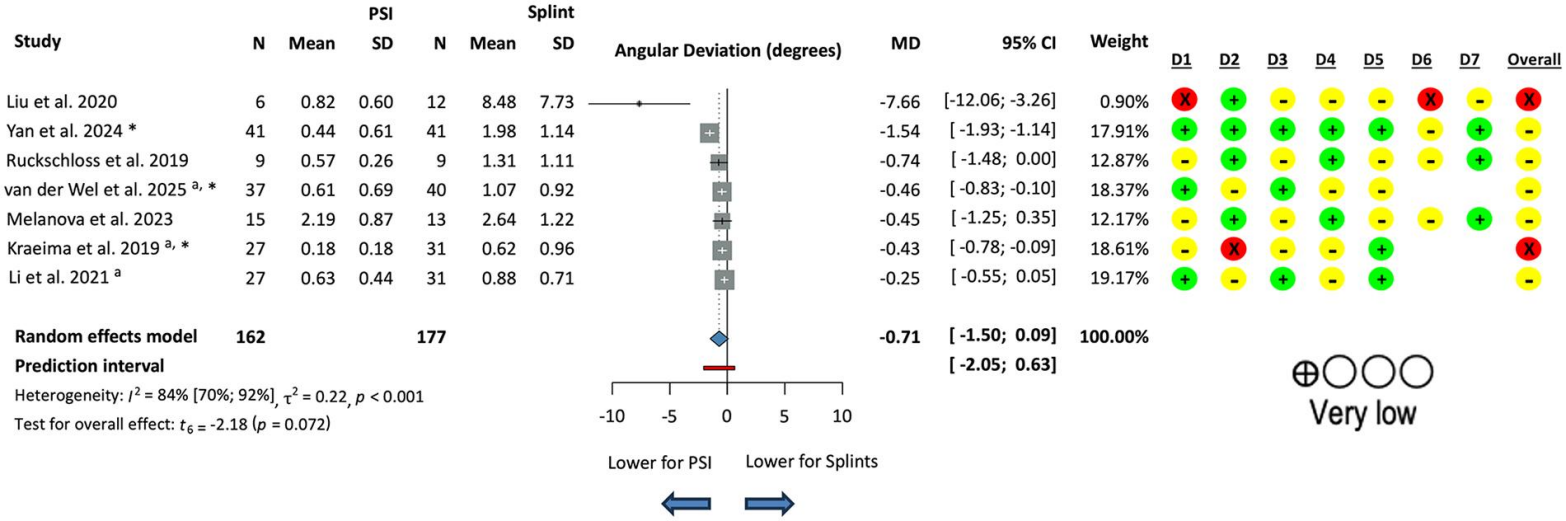
Forest plot demonstrating the effect of PSI vs. splint-based approaches in LFI osteotomies on the mean deviation from the planned position (in degrees) in yaw. *N*, number of patients in each group; MD, mean difference; SD, standard deviation; CI, confidence interval; PSI, patient-specific implants. Studies marked with “*” indicate that means and/or standard deviations were estimated from medians, quartiles, or ranges. Studies marked with “a” are RCTs. Certainty of evidence: very low.

**Figure 7:**
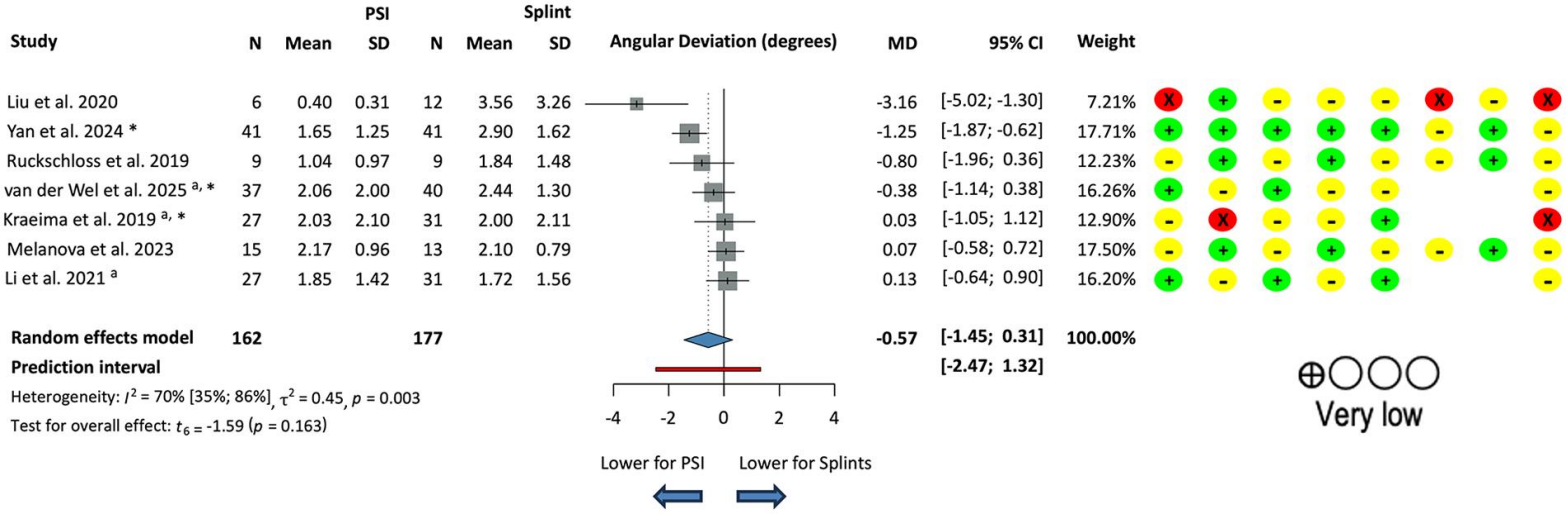
Forest plot demonstrating the effect of PSI vs. splint-based approaches in LFI osteotomies on the mean deviation from the planned position (in degrees) in pitch. *N*, number of patients in each group; MD, mean difference; SD, standard deviation; CI, confidence interval; PSI, patient-specific implants. Studies marked with “*” indicate that means and/or standard deviations were estimated from medians, quartiles, or ranges. Studies marked with “a” are RCTs. Certainty of evidence: very low.

We found no significant difference in yaw and pitch. However, surgical accuracy was consistently improved across studies, with a pooled MD of (MD = −0.71°; 95% CI [−1.50; 0.09]) in yaw (Figure 6) and (MD = −0.57°; 95% CI [−1.45; 0.31]) in pitch (Figure 7).

### 3.4 Secondary outcomes

While the primary focus of the present study was the quantitative assessment of surgical accuracy, some secondary outcomes were also systematically reviewed in LFI osteotomies, due to limited available data (Table 1).

**Table 1:** Summary of secondary outcomes not quantitatively pooled due to limited data.

Outcome	Key findings	Limitations of available evidence
Safety outcomes (accidents, complications, blood loss)	No significant differences in terms of complication rates and blood loss.	Mostly retrospective cohorts.
		Complication definitions vary across studies.
	Fewer dental root injuries with PSI compared to splints.	Rare events may be under-reported.
Stability	PSI may improve long-term stability, particularly in cleft cases, while other studies found no difference (<1 mm relapse at 1 year).	Mostly retrospective cohorts.
		Heterogeneity in terms of populations.
Operative time	Reduction of operative time in splintless OS.	This can depend on surgeon experience and workflow.
		Few standardized comparative studies.
Surgeon satisfaction	Higher reported satisfaction with PSI workflow.	Based on subjective assessment without validated scoring systems.
		Limited data available.
Cost-effectiveness	No studies were identified.	No formal economic analysis exists in the literature.
		Statements rely on indirect or estimated cost metrics.
		Evidence based on narrative reviews and small cohorts.

PSI, patient-specific implants; OS, orthognathic surgery; DST, digital surgical templates.

#### 3.4.1 3D global geometric deviation

Melanova et al. found no significant difference in 3D deviation between PSI (0.60 mm, 95% CI [0.46; 0.74]) and splints (0.86 mm, 95% CI [0.44; 1.28]) in nonsegmental LFI osteotomies (52). In contrast, Cassoni et al. found a significant difference (*p* = 0.02) with a lower mean 3D deviation in the PSI group (0.919 mm, SD = 0.062) compared to the splint group (1.783 mm, SD = 0.31) (54).

#### 3.4.2 Accidents, complications, and blood loss

Li et al. reported no significant difference in serious complications: infection occurred in 3.7% of the PSI group (1 of 27 patients) and 3.2% of the splint group (1 of 31 patients). No significant difference was found in intraoperative blood loss: median volume was 600 mL (IQR 600–800 mL) in the PSI group and 650 mL (IQR 500–800 mL) in the splint group (46). Similarly, Fleury et al. found no significant difference in overall or individual complications, including hematoma, seroma, infection, unplanned return to surgery, hardware removal, and need for revision (55).

A retrospective study of 126 LFI cases (2010–2018) assessed CT-based dental injuries from osteosynthesis screws. Among 61 patients treated with the splint-based technique and conventional plates, 10 injuries were found in 8 patients. In contrast, no injuries were reported in the 65 PSI cases (*p* < 0.001) (56).

#### 3.4.3 Stability

Long-term stability is essential in LFI osteotomies. Van der Wel et al. found no significant difference between PSI and virtually planned splints with conventional plates, showing <1 mm and <1° relapse at 1 year (57). In contrast, in a cohort of 73 cleft patients, Varidel et al. reported relapse >1 mm in only 5% of PSI cases, compared to >50% with splints and conventional plates (58), suggesting that PSI may offer greater stability in cleft cases. However, data remain limited.

#### 3.4.4 Surgeon satisfaction

Kraeima et al. assessed surgeon satisfaction with the intervention and reported that the PSI method was rated 7.8 out of 10 based on their experience, compared to conventional techniques (45).

#### 3.4.5 Cost-effectiveness

No studies directly comparing the cost-effectiveness of PSI, DST, and splint-based approaches were identified.

### 3.5 Risk of bias assessment

The results of the risk of bias (RoB) assessment are displayed alongside forest plots for each outcome (Figures 2–7). Among the observational studies, we identified a serious risk in the study by Liu et al. (50) due to concerns about yaw and pitch measurements. The others were evaluated as having a moderate risk of bias, mainly because of intervention classification and outcome measurement issues (49, 51–53). For RCTs, Li et al., Chen et al., and van der Wel et al. raised some concerns about randomization and deviations from protocol (46–48). We observed a high risk of bias in the study by Kraeima et al. due to randomization flaws, missing data, and significant deviations from the intended interventions (45). A sensitivity analysis excluding Liu et al. (50) due to a serious risk of bias and inclusion of hemifacial microsomia patients, reduced heterogeneity, and yielded significant results in yaw (MD = −0.64; 95% CI [−1.15; −0.14]). Forest plots are shown in the Supplementary Material (SDC, Figures S1–S6).

### 3.6 Publication bias and heterogeneity

Egger's test and funnel plots were not interpretable due to the small number of studies (nine for linear and seven for angular accuracy) (SDC, Figures S7–S12). Interstudy heterogeneity varied, likely due to differences in design, sample size, and follow-up. Given the limited data, heterogeneity estimates should be interpreted with caution, although high heterogeneity did not affect the significance of the results.

## 4 Discussion

### 4.1 Main outcomes

Our findings can be summarized in three main points. First, splintless osteotomies appear to improve surgical accuracy compared to the splint-based technique, with the most significant differences observed in the anteroposterior direction (∼0.5 mm) and yaw angular accuracy (∼0.7°). Second, although statistical significance was not achieved in the DST subgroup due to limited data, the effect is promising, as surgical accuracy improved with DST. While PSI-based approaches appeared to be more accurate than DST, the findings should be interpreted cautiously. The number of included studies per subgroup was limited, and confidence intervals overlapped for translational accuracy outcomes. Both studies in the DST subgroup were conducted by the same research group (Chen et al.), although they included distinct patient populations under different study designs. Moreover, these studies employed an identical two-step bone-supported template design consisting of cutting and repositioning guides with screw-hole matching, which may not fully represent the broader spectrum of DST designs. Therefore, these findings should be regarded as exploratory and hypothesis-generating rather than definitive evidence of superiority, given the moderate to very low certainty of evidence. Third, both techniques generally remained within the clinical threshold of 2 mm, although the splint group showed higher mean discrepancies and larger standard deviations, potentially increasing the risk of clinically relevant errors and underscoring the importance of considering standard deviations alongside mean values (59, 60). Our findings align with those of Cho et al., who reported significantly fewer deviations of more than 2 mm with DST than with virtually planned splints (61). Sensitivity analysis excluding Liu et al., a study with high risk of bias and unclear reporting, allowed the overall yaw findings to become statistically significant (MD = −0.64°, 95% CI [−1.15; −0.14]) (SDC, Figure S6) (50).

The literature shows inconsistency regarding the clinically acceptable threshold for rotational movements, with some studies using a cut-off value of 2° (48, 62), whereas others accept values up to 4° (63–65). We adhered to the 2° threshold, recognizing that any improvement in accuracy can enhance surgical predictability.

The overall improvements observed in splintless OS (∼0.3–0.5 mm and ∼0.5°–0.7°) appear to be modest and may not translate into clear clinical benefits in all cases. Nevertheless, these differences indicate an enhanced surgical precision, further reducing the postoperative deviation below the commonly accepted thresholds of 2 mm and 2°. Although a formal consensus has not been established, several studies suggest that a 1 mm threshold could be used for accuracy assessment, which further supports the clinical relevance of our findings (66, 67). Notably, such improvements are particularly relevant in symmetry-critical planes, as many studies have reported deviations greater than 1 mm along the *x*- and *z*-axes (45–47, 49–51). One of the included randomized trials reported accuracy at the individual patient level, using predefined accuracy thresholds (optimal: <1 mm and <2°; good: 1–2 mm and <2°; suboptimal: >2 mm and >2°) (48). Optimal or good outcomes were achieved in 59.5% (22/37) in PSI group compared with 17.5% (7/40) patients in the CAD/CAM splint group (48). These findings suggest that threshold-based analyses may better capture clinically meaningful differences and the risk of clinically unacceptable outcomes than mean values alone.

Surgical sequencing in splint-based osteotomies is widely debated (68), although recent meta-analyses found similar results for maxilla-first and mandible-first approaches (69, 70). The mandible-first sequence may help in cases with large counterclockwise rotations and class II patients with unstable condyles (71). However, two studies included in our quantitative analysis showed that PSI provided more accurate maxillary positioning than the mandible-first splint-based technique (48, 53).

Interpretation of pooled estimates should also consider clinical and methodological heterogeneity across the included studies. Variability existed in surgical protocols, including differences in operative sequencing. Two studies used mandible-first approaches in the CAD/CAM splints group (48, 53), whereas the remaining studies employed maxilla-first workflows. Considerable variability also existed in virtual planning and postoperative assessment workflows, including different planning software (e.g., 3-Matic, Geomagic, Maxilim, and CloudCompare), registration techniques (e.g., voxel- or surface-based), landmark definitions, and measurement approaches. (SDC, Table S1) Seven studies performed surface-based registration (46, 47, 49–53), whereas only two studies used voxel-based registration (45, 48). Although Almukhtar et al. found no significant differences between methods (0.047 mm in voxel vs. 0.05 mm in surface), surface-based registration demonstrated greater variability, possibly due to the additional 3D rendering step required prior to registration, which may introduce further sources of error (72). Although PSI and DST are both considered splintless techniques, they should not be regarded as interchangeable interventions. While PSI incorporates patient-specific fixation hardware designed to directly transfer and maintain the virtual plan, DST primarily serves as a transfer tool and relies on subsequent fixation with stock osteosynthesis plates. In addition, differences in patient populations may have contributed to clinical heterogeneity, as one study included patients with hemifacial microsomia, which is often associated with greater baseline asymmetry and increased surgical complexity (50). Evidence on the use of PSI for mandibular repositioning is limited. One study found no significant difference between PSI and splint-based techniques, with both methods staying within clinical limits (<2 mm, <4°). PSI led to slightly lower mean rotational discrepancies, but also showed the largest individual deviation, indicating high variability (29). However, evidence is limited.

Diaconu et al. conducted a meta-analysis and reported statistically significant improvements in translational and rotational accuracy with PSI compared to splints in LFI osteotomies (73). However, certain methodological limitations are noteworthy, including no prospectively registered protocol, lack of sensitivity analysis, and limited bias assessment. In contrast, our study includes more data, three additional studies in the PSI subgroup (48, 52, 53), and compares DST with splints as a subgroup analysis (47, 51).

### 4.2 Secondary outcomes

Beyond accuracy, surgical decision-making also depends on safety outcomes, stability, operative efficiency, surgeon satisfaction, and cost-effectiveness. The evidence for these secondary outcomes was limited. Most included studies reported comparable complication rates, blood loss, and stability between PSI and splint-based approaches. These findings align with other studies reporting low postoperative complication rates with PSI in LFI and BSSO procedures, supporting its safety profile (74, 75). Neurosensory disturbance of the inferior alveolar nerve is among the most common complications following BSSO (up to 85%). Gennaro et al. demonstrated that greater intraoperative nerve exposure is associated with increased postoperative neurosensory impairment (76). In splintless workflows, osteotomy lines and screw positioning are planned with consideration of the nerve trajectory, which may help reduce unfavorable splits and nerve injury. Previous studies have reported no persistent paresthesia in splintless cohorts, whereas isolated cases were observed in the CAD/CAM splints group (77, 78). Minimally invasive BSSO has also gained attention, but its true impact on nerve morbidity remains uncertain, and more research is warranted (79). Regarding stability, two other studies demonstrated relapse of less than 1 mm and 1° at skeletal and dental reference points, with follow-up periods ranging from 6 to 34 months (80, 81). Regarding operative efficiency, the literature suggests that the splintless workflow may reduce preoperative planning time and shorten intraoperative time in the operating theatre (55, 82). Similarly, in a UK survey, 61% of surgeons reported that splintless approaches reduce operative time by avoiding intraoperative measurements, plate bending, and intermaxillary fixation (83). Direct cost-effectiveness comparisons between splintless OS and virtually planned splints are not available. Mazzoni et al. (2015) first reported using PSI for upper maxillary repositioning at approximately €750 per case (84). Heufelder et al. (2017) suggested that PSI could replace CAD/CAM splints in routine practice (14); however, later studies acknowledged cost as a barrier, although no direct economic evaluations were available (46, 49, 53, 85–87). Despite the potential benefits, PSI cost-effectiveness remains unclear due to limited high-quality data. Conversely, splintless OS may reduce overall cost by shortening planning and surgery time (82). Lin et al. estimated that the average price of DST is approximately 200 USD (88), a factor that may encourage their adoption instead of PSI.

### 4.3 Implications for practice and research

Our results suggest that PSI or DST may be considered in selected complex cases to achieve more predictable results. Although cost barriers may still exist, this may be overcome by the clinical advantages of splintless OS. DST may represent a potentially lower-cost alternative, providing nearly the same level of accuracy and clinical benefits.

Larger prospective comparative studies are needed to assess long-term stability, soft-tissue response, and patient-reported outcomes, including aesthetic satisfaction and quality of life, as well as functional outcomes such as temporomandibular joint dysfunction, masticatory function, and airway function. Temporomandibular joint outcomes should include preoperative screening, based on which patients should be stratified, and postoperative assessments of mandibular range of motion, maximal mouth opening, pain, joint sounds, palpation findings, and imaging when clinically indicated (89). Masticatory function should be evaluated using objective measures such as comminution tests, bite force, and occlusal contact area, ideally at 6 months and 1–2 years postoperatively (90). Airway outcomes may be assessed using CBCT-derived posterior airway space and minimal axial area measurements preoperatively, in the early postoperative period, and at least 1 year after surgery (91). Patient-centered outcomes should be collected using validated patient-reported outcome measures, such as OQLQ, FACE-Q, and satisfaction VAS, preferably after postoperative orthodontic treatment is completed or at least 6 months postoperatively (92, 93). Future studies should also evaluate cost-effectiveness, operative time, blood loss, and complications. Evidence for DST remains limited, and well-designed randomized controlled trials directly comparing DST with PSI and with splint-based techniques are particularly needed. Additionally, more robust comparative studies investigating splintless mandibular repositioning in BSSO are needed. The potential effect of novel PSI designs used in minimally invasive OS on surgical accuracy and patient satisfaction should also be investigated.

Policymakers should focus on developing clear guidelines, while insurance companies should support the selective use of PSI or DST.

### 4.4 Strengths and limitations

The strengths of our study include a preregistered protocol, rigorous methods, adherence to Cochrane and PRISMA standards, subgroup and sensitivity analyses. To our knowledge, this is the largest patient cohort to date, expanding and improving the methodological rigor of the only previous meta-analysis on this topic (73).

Limitations include the small number of eligible studies and heterogeneity in study design and methodology. Importantly, most studies assessed surgical accuracy within the early postoperative period (3–14 days), which reflects initial positioning but does not capture long-term skeletal stability. An additional limitation is the absence of comparative patient-centered outcomes such as aesthetic satisfaction, quality of life, and functional outcomes. Although our initial scope included both LFI and BSSO osteotomies, only one eligible study reporting BSSO accuracy was identified. Additionally, data on secondary outcomes in LFI were insufficient to support a quantitative synthesis. Another limitation is the moderate to high risk of bias observed in some domains.

### 4.5 Conclusion

The present study suggests that the splintless approach may improve accuracy in non-segmental Le Fort I osteotomies. PSI appeared to show the largest improvements in translational movements along the anteroposterior axis and for rotational accuracy around the vertical axis (yaw). However, given the moderate to very low certainty of evidence, these findings should be interpreted with caution. Given the limited number of studies in the DST subgroup, these observations should be regarded as exploratory and hypothesis-generating, rather than definitive evidence of superiority.

## Data Availability

The original contributions presented in the study are included in the article/Supplementary Material, further inquiries can be directed to the corresponding author.
