# Supplementary material for: Splintless approach improves surgical accuracy in Le Fort I osteotomies: a systematic review and meta-analysis
Source: Front Oral Health. 2026 Jun 16;7:1854976. doi: 10.3389/froh.2026.1854976 (PMC13316659; doi:10.3389/froh.2026.1854976)
Supplement: Supplementary file 1 [file Supplementaryfile1.pdf]

frontiers

Supplementary Material

Splintless approach improves surgical accuracy in Lefort I osteotomies: A systematic review and meta-analysis

Sandu D. et al. (2026). Front Oral Health

Table of contents

1. SDC, Methods 1. PRISMA checklist
2. SDC, Methods 2. AMSTAR 2 checklist
3. SDC, Methods 3. Detailed search key
4. SDC, Methods 4. GradePRO summary table for certainty of evidence
5. SDC, Table S1. Baseline characteristics of included studies in the quantitative analysis
6. SDC, Table S2. Baseline characteristics of included studies in the systematic review
7. SDC, Figure S1. Forest plot (excluding the study by Liu et al.[7]) demonstrating the effect of splintless (PSI/DST) versus splint-based approaches in LFI osteotomies on the mean deviation from the planned position (in mm) in the anteroposterior axis.
8. SDC, Figure S2. Forest plot (excluding the study by Liu et al.[7]) demonstrating the effect of splintless (PSI/DST) versus splint-based approaches in LFI osteotomies on the mean deviation from the planned position (in mm) in the mediolateral axis.
9. SDC, Figure S3. Forest plot (excluding the study by Liu et al.[7]) demonstrating the effect of splintless (PSI/DST) versus splint-based approaches in LFI osteotomies on the mean deviation from the planned position (in mm) in the vertical axis.
10. SDC, Figure S4. Forest plot (excluding the study by Liu et al.[7]) demonstrating the effect of splintless (PSI) versus splint-based approaches in LFI osteotomies on the mean deviation from the planned position (in degrees) in roll.
11. SDC, Figure S5. Forest plot (excluding the study by Liu et al.[7]) demonstrating the effect of splintless (PSI) versus splint-based approaches in LFI osteotomies on the mean deviation from the planned position (in degrees) in pitch.
12. SDC, Figure S6. Forest plot (excluding the study by Liu et al. [7]) demonstrating the effect of splintless (PSI) versus splint-based approaches in LFI osteotomies on the mean deviation from the planned position (in degrees) in yaw.
13. SDC, Figure S7. Funnel plot assessing the publication bias for the mean deviation from the planned position in the anteroposterior axis.
14. SDC, Figure S8. Funnel plot assessing the publication bias for the mean deviation from the planned position in the mediolateral axis.
15. SDC, Figure S9. Funnel plot assessing the publication bias for the mean deviation from the planned position in the vertical axis.
16. SDC, Figure S10. Funnel plot assessing the publication bias for the mean deviation from the planned position in roll.
17. SDC, Figure S11. Funnel plot assessing the publication bias for the mean deviation from the planned position in pitch.
18. SDC, Figure S12. Funnel plot assessing the publication bias for the mean deviation from the planned position in yaw.

SDC, Methods 1. PRISMA 2020 checklist.

Section and Topic	Item #	Checklist item	Location where item is reported
**TITLE**			
Title	1	Identify the report as a systematic review.	p. 01
**ABSTRACT**			
Abstract	2	See the PRISMA 2020 for Abstracts checklist.	p. 01
**INTRODUCTION**			
Rationale	3	Describe the rationale for the review in the context of existing knowledge.	p. 02
Objectives	4	Provide an explicit statement of the objective(s) or question(s) the review addresses.	p. 02
**METHODS**			
Eligibility criteria	5	Specify the inclusion and exclusion criteria for the review and how studies were grouped for the syntheses.	p. 02
Information sources	6	Specify all databases, registers, websites, organisations, reference lists and other sources searched or consulted to identify studies. Specify the date when each source was last searched or consulted.	p. 02
Search strategy	7	Present the full search strategies for all databases, registers and websites, including any filters and limits used.	SDC, Methods 3
Selection process	8	Specify the methods used to decide whether a study met the inclusion criteria of the review, including how many reviewers screened each record and each report retrieved, whether they worked independently, and if applicable, details of automation tools used in the process.	p. 02
Data collection process	9	Specify the methods used to collect data from reports, including how many reviewers collected data from each report, whether they worked independently, any processes for obtaining or confirming data from study investigators, and if applicable, details of automation tools used in the process.	p. 02
Data items	10a	List and define all outcomes for which data were sought. Specify whether all results that were compatible with each outcome domain in each study were sought (e.g. for all measures, time points, analyses), and if not, the methods used to decide which results to collect.	p. 02
	10b	List and define all other variables for which data were sought (e.g. participant and intervention characteristics, funding sources). Describe any assumptions made about any missing or unclear information.	p. 02-03
Study risk of bias assessment	11	Specify the methods used to assess risk of bias in the included studies, including details of the tool(s) used, how many reviewers assessed each study and whether they worked independently, and if applicable, details of automation tools used in the process.	p. 03
Effect measures	12	Specify for each outcome the effect measure(s) (e.g. risk ratio, mean difference) used in the synthesis or presentation of results.	p. 03
Synthesis methods	13a	Describe the processes used to decide which studies were eligible for each synthesis (e.g. tabulating the study intervention characteristics and comparing against the planned groups for each synthesis (item #5)).	p. 03
	13b	Describe any methods required to prepare the data for presentation or synthesis, such as handling of missing summary statistics, or data conversions.	p. 03
	13c	Describe any methods used to tabulate or visually display results of individual studies and syntheses.	p. 03

Section and Topic	Item #	Checklist item	Location where item is reported
	13d	Describe any methods used to synthesize results and provide a rationale for the choice(s). If meta-analysis was performed, describe the model(s), method(s) to identify the presence and extent of statistical heterogeneity, and software package(s) used.	p. 03
	13e	Describe any methods used to explore possible causes of heterogeneity among study results (e.g. subgroup analysis, meta-regression).	p. 03
	13f	Describe any sensitivity analyses conducted to assess robustness of the synthesized results.	p. 03
Reporting bias assessment	14	Describe any methods used to assess risk of bias due to missing results in a synthesis (arising from reporting biases).	Not applicable
Certainty assessment	15	Describe any methods used to assess certainty (or confidence) in the body of evidence for an outcome.	p. 03
**RESULTS**			
Study selection	16a	Describe the results of the search and selection process, from the number of records identified in the search to the number of studies included in the review, ideally using a flow diagram.	p. 03
	16b	Cite studies that might appear to meet the inclusion criteria, but which were excluded, and explain why they were excluded.	Figure 1
Study characteristics	17	Cite each included study and present its characteristics.	p. 03, SDC Table S1, SDC Table S2
Risk of bias in studies	18	Present assessments of risk of bias for each included study.	p. 07-08, Figures 2-7
Results of individual studies	19	For all outcomes, present, for each study: (a) summary statistics for each group (where appropriate) and (b) an effect estimate and its precision (e.g. confidence/credible interval), ideally using structured tables or plots.	Figures 2-7
Results of syntheses	20a	For each synthesis, briefly summarise the characteristics and risk of bias among contributing studies.	p. 07-08, Figures 2-7
	20b	Present results of all statistical syntheses conducted. If meta-analysis was done, present for each the summary estimate and its precision (e.g. confidence/credible interval) and measures of statistical heterogeneity. If comparing groups, describe the direction of the effect.	p. 03-08, Figures 2-7
	20c	Present results of all investigations of possible causes of heterogeneity among study results.	p. 08, 09
	20d	Present results of all sensitivity analyses conducted to assess the robustness of the synthesized results.	p. 08, SDC Figures S1-6
Reporting biases	21	Present assessments of risk of bias due to missing results (arising from reporting biases) for each synthesis assessed.	Not applicable
Certainty of evidence	22	Present assessments of certainty (or confidence) in the body of evidence for each outcome assessed.	p. 03-08, Figures 2-7, SDC Methods 4
**DISCUSSION**			
Discussion	23a	Provide a general interpretation of the results in the context of other evidence.	p. 08-09
	23b	Discuss any limitations of the evidence included in the review.	p. 10

Section and Topic	Item #	Checklist item	Location where item is reported
	23c	Discuss any limitations of the review processes used.	p. 10
	23d	Discuss implications of the results for practice, policy, and future research.	p. 09-10
**OTHER INFORMATION**			
Registration and protocol	24a	Provide registration information for the review, including register name and registration number, or state that the review was not registered.	p. 02
	24b	Indicate where the review protocol can be accessed, or state that a protocol was not prepared.	p. 02
	24c	Describe and explain any amendments to information provided at registration or in the protocol.	p. 02
Support	25	Describe sources of financial or non-financial support for the review, and the role of the funders or sponsors in the review.	p. 10
Competing interests	26	Declare any competing interests of review authors.	p. 10
Availability of data, code and other materials	27	Report which of the following are publicly available and where they can be found: template data collection forms; data extracted from included studies; data used for all analyses; analytic code; any other materials used in the review.	Available upon request

frontiers

SDC, Methods 2. AMSTAR 2 checklist: a critical appraisal tool for systematic reviews that include randomised or non-randomised studies of healthcare interventions, or both.

1. Did the research questions and inclusion criteria for the review include the components of PICO?

For Yes:
- ☐ Population
- ☐ Intervention
- ☐ Comparator group
- ☐ Outcome

Optional (recommended)
- ☐ Timeframe for follow-up

- ☑ Yes
- ☐ No

2. Did the report of the review contain an explicit statement that the review methods were established prior to the conduct of the review and did the report justify any significant deviations from the protocol?

For Partial Yes:
The authors state that they had a written protocol or guide that included ALL the following:
- ☐ review question(s)
- ☐ a search strategy
- ☐ inclusion/exclusion criteria
- ☐ a risk of bias assessment

For Yes:
As for partial yes, plus the protocol should be registered and should also have specified:
- ☐ a meta-analysis/synthesis plan, if appropriate, *and*
- ☐ a plan for investigating causes of heterogeneity
- ☐ justification for any deviations from the protocol

- ☑ Yes
- ☐ Partial Yes
- ☐ No

3. Did the review authors explain their selection of the study designs for inclusion in the review?

For Yes, the review should satisfy ONE of the following:
- ☐ *Explanation for* including only RCTs
- ☐ OR *Explanation for* including only NRSI
- ☑ OR *Explanation for* including both RCTs and NRSI

- ☑ Yes
- ☐ No

4. Did the review authors use a comprehensive literature search strategy?

For Partial Yes (all the following):
- ☑ searched at least 2 databases (relevant to research question)
- ☑ provided key word and/or search strategy
- ☑ justified publication restrictions (e.g. language)

For Yes, should also have (all the following):
- ☑ searched the reference lists / bibliographies of included studies
- ☑ searched trial/study registries
- ☑ included/consulted content experts in the field
- ☐ where relevant, searched for grey literature
- ☑ conducted search within 24 months of completion of the review

- ☐ Yes
- ☑ Partial Yes
- ☐ No

5. Did the review authors perform study selection in duplicate?

For Yes, either ONE of the following:
- ☐ at least two reviewers independently agreed on selection of eligible studies and achieved consensus on which studies to include
- ☑ OR two reviewers selected a sample of eligible studies and achieved good agreement (at least 80 percent), with the remainder selected by one reviewer.

- ☑ Yes
- ☐ No

6. Did the review authors perform data extraction in duplicate?

For Yes, either ONE of the following:<br>☐ at least two reviewers achieved consensus on which data to extract from included studies<br>☑ OR two reviewers extracted data from a sample of eligible studies <u>and</u> achieved good agreement (at least 80 percent), with the remainder extracted by one reviewer.	☑ Yes<br>☐ No

7. Did the review authors provide a list of excluded studies and justify the exclusions?

For Partial Yes:<br>☐ provided a list of all potentially relevant studies that were read in full-text form but excluded from the review	For Yes, must also have:<br>☐ Justified the exclusion from the review of each potentially relevant study	☐ Yes<br>☐ Partial Yes<br>☑ No

8. Did the review authors describe the included studies in adequate detail?

For Partial Yes (ALL the following):<br>☐ described populations<br>☐ described interventions<br>☐ described comparators<br>☐ described outcomes<br>☐ described research designs	For Yes, should also have ALL the following:<br>☐ described population in detail<br>☐ described intervention in detail (including doses where relevant)<br>☐ described comparator in detail (including doses where relevant)<br>☐ described study's setting<br>☐ timeframe for follow-up	☑ Yes<br>☐ Partial Yes<br>☐ No

9. Did the review authors use a satisfactory technique for assessing the risk of bias (RoB) in individual studies that were included in the review?

**RCTs**<br>For Partial Yes, must have assessed RoB from<br>☐ unconcealed allocation, *and*<br>☐ lack of blinding of patients and assessors when assessing outcomes (unnecessary for objective outcomes such as all-cause mortality)	For Yes, must also have assessed RoB from:<br>☐ allocation sequence that was not truly random, *and*<br>☐ selection of the reported result from among multiple measurements or analyses of a specified outcome	☑ Yes<br>☐ Partial Yes<br>☐ No<br>☐ Includes only NRSI
**NRSI**<br>For Partial Yes, must have assessed RoB:<br>☐ from confounding, *and*<br>☐ from selection bias	For Yes, must also have assessed RoB:<br>☐ methods used to ascertain exposures and outcomes, *and*<br>☐ selection of the reported result from among multiple measurements or analyses of a specified outcome	☑ Yes<br>☐ Partial Yes<br>☐ No<br>☐ Includes only RCTs

10. Did the review authors report on the sources of funding for the studies included in the review?

For Yes<br>☐ Must have reported on the sources of funding for individual studies included in the review. Note: Reporting that the reviewers looked for this information but it was not reported by study authors also qualifies	☐ Yes<br>☑ No

11. If meta-analysis was performed did the review authors use appropriate methods for statistical combination of results?

RCTs
For Yes:

- ☐ The authors justified combining the data in a meta-analysis
  - ☐ AND they used an appropriate weighted technique to combine study results and adjusted for heterogeneity if present.
  - ☐ AND investigated the causes of any heterogeneity

☑ Yes
☐ No
☐ No meta-analysis conducted

For NRSI
For Yes:

- ☑ The authors justified combining the data in a meta-analysis
  - ☑ AND they used an appropriate weighted technique to combine study results, adjusting for heterogeneity if present
  - ☑ AND they statistically combined effect estimates from NRSI that were adjusted for confounding, rather than combining raw data, or justified combining raw data when adjusted effect estimates were not available
  - ☐ AND they reported separate summary estimates for RCTs and NRSI separately when both were included in the review

☐ Yes
☑ No
☐ No meta-analysis conducted

12. If meta-analysis was performed, did the review authors assess the potential impact of RoB in individual studies on the results of the meta-analysis or other evidence synthesis?

For Yes:

- ☐ included only low risk of bias RCTs
- ☑ OR, if the pooled estimate was based on RCTs and/or NRSI at variable RoB, the authors performed analyses to investigate possible impact of RoB on summary estimates of effect.

☑ Yes
☐ No
☐ No meta-analysis conducted

13. Did the review authors account for RoB in individual studies when interpreting/ discussing the results of the review?

For Yes:

- ☐ included only low risk of bias RCTs
- ☑ OR, if RCTs with moderate or high RoB, or NRSI were included the review provided a discussion of the likely impact of RoB on the results

☑ Yes
☐ No

14. Did the review authors provide a satisfactory explanation for, and discussion of, any heterogeneity observed in the results of the review?

For Yes:

- ☐ There was no significant heterogeneity in the results
- ☑ OR if heterogeneity was present the authors performed an investigation of sources of any heterogeneity in the results and discussed the impact of this on the results of the review

☑ Yes
☐ No

15. If they performed quantitative synthesis did the review authors carry out an adequate investigation of publication bias (small study bias) and discuss its likely impact on the results of the review?

For Yes:

- ☐ performed graphical or statistical tests for publication bias and discussed the likelihood and magnitude of impact of publication bias

×
☑ Yes
☐ No
☐ No meta-analysis conducted

16. **Did the review authors report any potential sources of conflict of interest, including any funding they received for conducting the review?**	
For Yes:<br>☑ The authors reported no competing interests OR<br>☐ The authors described their funding sources and how they managed potential conflicts of interest	☑ Yes<br>☐ No

SDC, Methods 3. – Detailed search key.

A. PubMed

Number of results: 3732
Searchkey:

(((“orthognathic” OR “single jaw” OR “double jaw” OR “Bilateral sagittal split” OR “Maxillary”) AND (“surgery” OR “osteotomy” OR “treatment” OR “realignment” OR “operation”)) OR “Le Fort I” OR “Le Fort 1” OR “BSSO”)
AND
((“patient-specific” OR "patient specific" OR custom* OR personal* OR “splintless” OR “waferless” OR “pre-bent” OR “prebent”) AND (implant* OR “osteosynthesis” OR plate* OR “fixation device”) OR “PSI” OR template* OR guide*)

A. Embase

Number of results: 4300
Searchkey:

(((orthognathic OR “single jaw” OR “double jaw” OR “Bilateral sagittal split” OR Maxillary) AND (surgery OR osteotomy OR treatment OR realignment OR operation)) OR “Le Fort I” OR “Le Fort 1” OR BSSO)
AND
((patient-specific OR "patient specific" OR custom* OR personal* OR splintless OR waferless OR pre-bent OR prebent) AND (implant* OR osteosynthesis OR plate* OR “fixation device”) OR PSI OR template* OR guide*)

B. Central (Cochrane)

Number of results: 531
Searchkey:

(((orthognathic OR “single jaw” OR “double jaw” OR “Bilateral sagittal split” OR Maxillary) AND (surgery OR osteotomy OR treatment OR realignment OR operation)) OR “Le Fort I” OR “Le Fort 1” OR BSSO)
AND
((patient-specific OR "patient specific" OR custom* OR personal* OR splintless OR waferless OR pre-bent OR prebent) AND (implant* OR osteosynthesis OR plate* OR “fixation device”) OR PSI OR template* OR guide*)

SDC, Methods 4. GRADEPro summary table for certainty of evidence in the PSI subgroup.

Patient-Specific Implants compared to Splints in Patients undergoing Orthognathic Surgery

Certainty assessment							№ of patients		Effect		Certainty	Importance
№ of studies	Study design	Risk of bias	Inconsistency	Indirectness	Imprecision	Other considerations	Patient-Specific Implants	Splints	Relative (95% CI)	Absolute (95% CI)		
**Linear accuracy along y-axis (follow-up: range 3 days to 21)**												
7	non-randomised studies[a]	serious[b,c]	not serious[d]	not serious[e]	not serious[f]	none	162	177	-	mean **0.51 mm lower** (0.82 lower to 0.19 lower)	⊕⊕⊕◯ Moderate[b,c,d,e,f]	IMPORTANT
**Linear accuracy along x-axis (follow-up: range 3 days to 21 days)**												
7	non-randomised studies[a]	serious[b,c]	serious[g]	not serious[e]	not serious[f]	none	162	177	-	mean **0.42 mm lower** (0.73 lower to 0.11 lower)	⊕⊕◯◯ Low[b,c,e,f,g]	IMPORTANT
**Linear accuracy along z-axis**												
7	non-randomised studies[a]	serious[b,c]	serious[g]	not serious[e]	serious[h]	none	162	177	-	mean **0.29 mm lower** (0.58 lower to 0)	⊕◯◯◯ Very low[b,c,e,g,h]	IMPORTANT
**Angular accuracy around y-axis (follow-up: range 3 days to 21 days)**												
7	non-randomised studies[a]	serious[b,c]	serious[g]	not serious[e]	not serious[f]	none	162	177	-	mean **0.48 degrees lower** (0.88 lower to 0.08 lower)	⊕⊕◯◯ Low[b,c,e,f,g]	IMPORTANT
**Angular accuracy around x-axis (follow-up: range 3 days to 21 days)**												
7	non-randomised studies[a]	serious[b,c]	serious[g]	not serious[e]	serious[h]	none	162	177	-	mean **0.57 degrees lower** (1.45 lower to 0.31 higher)	⊕◯◯◯ Very low[b,c,e,g,h]	IMPORTANT
**Angular accuracy around z-axis (follow-up: range 3 days to 21 days)**												

Certainty assessment							№ of patients		Effect		Certainty	Importance
№ of studies	Study design	Risk of bias	Inconsistency	Indirectness	Imprecision	Other considerations	Patient-Specific Implants	Splints	Relative (95% CI)	Absolute (95% CI)		
7	non-randomised studies[a]	very serious[b,c]	serious[g]	not serious[e]	serious[h]	none	162	177	-	mean **0.64 degrees lower** (1.15 lower to 0.14 lower)	⊕◯◯◯ Very low[b,c,e,f,g]	IMPORTANT

CI: confidence interval

Explanations

a. Of the 7 pooled studies, 3 were RCTs, and the remaining were observational studies

b. Observational studies were assessed with the ROBINS-I tool: Liu et al. - High risk of bias; Malenova et al. - Moderate risk of bias; Ruckschloss et al. - Moderate risk of bias; Yan et al. - Moderate risk of bias.

c. RCTs were assessed with the RoB2 tool: Li et al. - Some concerns; Kraeima et al. - High risk of bias; van der Wel - Some concerns

d. Visual consistency can be observed in the forest plot. The point estimates steadily favor patient-specific implants, showing lower mean deviation values.

e. The available evidence directly answers the clinical question.

f. Wide confidence interval likely due to heterogeneity

g. Visual consistency can be observed in the forest plot. However, high heterogeneity exists.

h. Wide confidence interval likely due to heterogeneity, but it includes the null value.

SDC, Table S1. Baseline characteristics of included studies in the quantitative analysis.

Author (year)	Study site	Study design	Number of analyzed patients (female %)	Age (year) ‡	Assessed outcome/s	Number of Patients in the intervnetion group	Number of patients in the control group	Virtual planning software	Outcome measurement
van der Wel et al. 2025[1]	Netherlands	RCT	77 (ND)	29 (±11)	Linear and angular accuracy	PSI: 37<br>Surgical sequence: maxilla-first	Splints: 40<br>Surgical sequence: mandible-first	ND	**Voxel-based registration** of pre- and postoperative CBCTs.<br>**Software**: ND.<br>**Landmarks**: upper incisor point.
Yan et al. 2024[2]	China	Observational	82 (85)	25.5 (ND)	Linear and angular accuracy	PSI: 41<br>Surgical sequence: maxilla-first	Splints: 41<br>Surgical sequence: mandible-first	Geomagic Studio (3D Systems, Rock Hill, SC, USA)	**Surface registration** of pre- and postoperative CBCTs.<br>**Software**: 3-Matic (Materialise, Leuven, Belgium).<br>**Landmarks**: UI, U6L, U6R.
Malenova et al. 2023[3]	Germany	Observational	28 (43)	27.5 (ND)	Linear and angular accuracy, 3D global geometric deviation	PSI: 15<br>Surgical sequence: maxilla-first	Splints: 13<br>Surgical sequence: maxilla-first	ProPlan CMF (Materialise, Leuven, Belgium)	**Surface registration** of pre- and postoperative CBCTs.<br>**Software**: 3-Matic (Materialise, Leuven, Belgium).<br>**Landmarks**: UI, U3L, U3R, U7L, U7R.
Chen et al. 2022[4]	China	Observational	70 (66)	23.1 (3.4)	Linear accuracy	DST: 37<br>Surgical sequence: maxilla-first	Splints: 33<br>Surgical sequence: maxilla-first	Mimics 19.0 and 3-Matic 11.0 (Materialise, Leuven, Belgium)	**Surface registration** of pre- and postoperative CBCTs.<br>**Software**: ND.<br>**Landmarks**: UI, U4L, U4R, U6L, U6R.
Chen et al. 2021[5]	China	RCT	41 (70)	23 (3)	Linear accuracy	DST: 20<br>Surgical sequence: maxilla-first	Splints: 21<br>Surgical sequence: maxilla-first	Mimics 19.0 and 3-Matic 11.0 (Materialise, Leuven, Belgium)	**Surface registration** of pre- and postoperative CBCTs.<br>**Software**: Geomagic Control 2015 (3D Systems, Rock Hill SC, USA).<br>**Landmarks**: UI, U4L, U4R, U6L, U6R.

BiaoLi et al. 2021[6]	China	RCT	58 (62)	23.7 (ND)	Linear and angular accuracy	PSI: 27 Surgical sequence: maxilla-first	Splints: 31 Surgical sequence: maxilla-first/mandible-first	ND	**Surface registration** of pre- and postoperative CBCTs. **Software**: 3-Matic (Materialise, Leuven, Belgium). **Landmarks**:UI, U6L, U6R.
Liu et al. 2020[7]	China	Observational	18 (50)	17-30 (ND)	Linear and angular accuracy	PSI: 6 Surgical sequence: maxilla-first	Splints: 12 Surgical sequence: maxilla-first	ProPlan 2.1, (Materialise, Leuven, Belgium)	**Surface registration** of pre- and postoperative CBCTs. **Software**: 3-Matic (Materialise, Leuven, Belgium). **Landmarks**: UI, U6L, U6R.
Kraeima et al. 2019[8]	Netherlands	RCT	58 (47)	28.5 (9)	Linear and angular accuracy	PSI: 27 Surgical sequence: maxilla-first	Splints: 31 Surgical sequence: maxilla-first	ND	**Voxel-based registration** of pre- and postoperative CBCTs. **Software**: Maxilim v2.3 (Medicim NV, Mechelen, Belgium). **Landmarks** UI, U6L, U6R.
Ruckschloss et al. 2019[9]	Germany	Observational	18 (39)	19.8 (2.19)	Linear and angular accuracy	PSI: 9	Splints: 9	IPS CaseDesigner (KLS Martin, Tuttlingen, Germany)	**Surface registration** of pre- and postoperative CBCTs. **Software**: CloudCompare (Telecom ParisTech, Paris, France). **No landmarks used. ICU (iterative closest point) algorithm**

UI- mesial contact point of the incisors; **U3L**- the tip of the left canine cusp; **U4L**- the tip of the bucal cusp of the left first premolar; **U4R**- the tip of the bucal cusp of the right first premolar **U3R**- the tip of the right canine cusp; **U6L**- the tip of the mesiobucal cusp of the left first molar; **U6R**- the tip of the mesiobucal cusp of the right first molar; **U7L**- the tip of the mesiobucal cusp of the left second molar; **U7R**- the tip of the mesiobucal cusp of the right first molar.

SDC, Table S2. Baseline characteristics of included studies in the systematic review.

Author (year)	Study site	Study design	Number of analyzed patients (female %)	Age (year) ‡	Outcome/s	Number of patients in the intervention group	Number of patients in the control group
Varidel et al. 2024[10]	Boston, Massachusetts	Observational	63 (52)	18.2 (±2.5)	Skeletal Stability of LFI in patients with clef lip and palate	PSI: 23	Splints and stock plates: 40
Cho et al. 2024[11]	Korea	Observational	33 (33)	24.2 (±6.2)	Accuracy of LFI (3D distance error, linear and angular deviations*)	DST: 17	Splints and stock plates: 16
Van der Wel er al. 2023[12]	Netherlands	RCT	27 (52)	27.7 (±8.3)	Skeletal Stability of LFI	PSI: 14	Splints and stock plates: 13
Pietzka et al. 2023[13]	Germany	Obsevational	126 (48.5)	24.9 (17.7-60.7)	Prevalence and localization of dental injuries in LFI caused by osteosyntesis screws	PSI: 65	Splints and stock plates: 61
Malenova et al. 2023[3]	Germany	Observational	28 (43)	27.5 (ND)	3D geometric deviation	PSI: 15	Splints and stock plates: 13
Harding et al. 2022[14]	Australia	Observational	55 (65)	27 (16-52)	Accuracy of proximal segment position after BSSO	PSI: 30	Splints and stock plates: 25
Fleury et al. 2022[15]	Washington, D.C.	Observational	43 (47)	23.1 (±7.8)	Postoperative complications Surgery time	PSI: 25	Splints and stock plates: 18

Cassoni et al. 2022[16]	Italy	Observational	6 (33)	29.7 (19-53)	3D global geometric deviation	PSI: 3	Splints and stock plates: 3
BiaoLi et al. 2021[6]	China	RCT	58 (62)	23.7 (ND)	Postoperative complications Blood loss	PSI: 27	Splints and stock plates: 31
Kraeima et al. 2019[8]	Netherlands	RCT	58 (47)	28.5 (9)	Suregon's satisfaction regarding surgery	PSI: 27	Splints and stock plates: 31

LFI- Lefort I osteotomy, **PSI**- Patient-specific implants, **DST**- Digital surgical templates, **RCT**- Randomized controlled clinical trial, **ND**- no data, **BSSO**- Bilateral sagittal split osteotomy.
The study marked with * provided signed values for linear and angular deviations, making it impossible to pool the data with the other studies.

SDC, Figure S1. Forest plot (excluding the study by Liu et al.[7]) demonstrating the effect of splintless (PSI/DST) versus splint-based approaches in LFI osteotomies on the mean deviation from the planned position (in mm) in the anteroposterior axis.

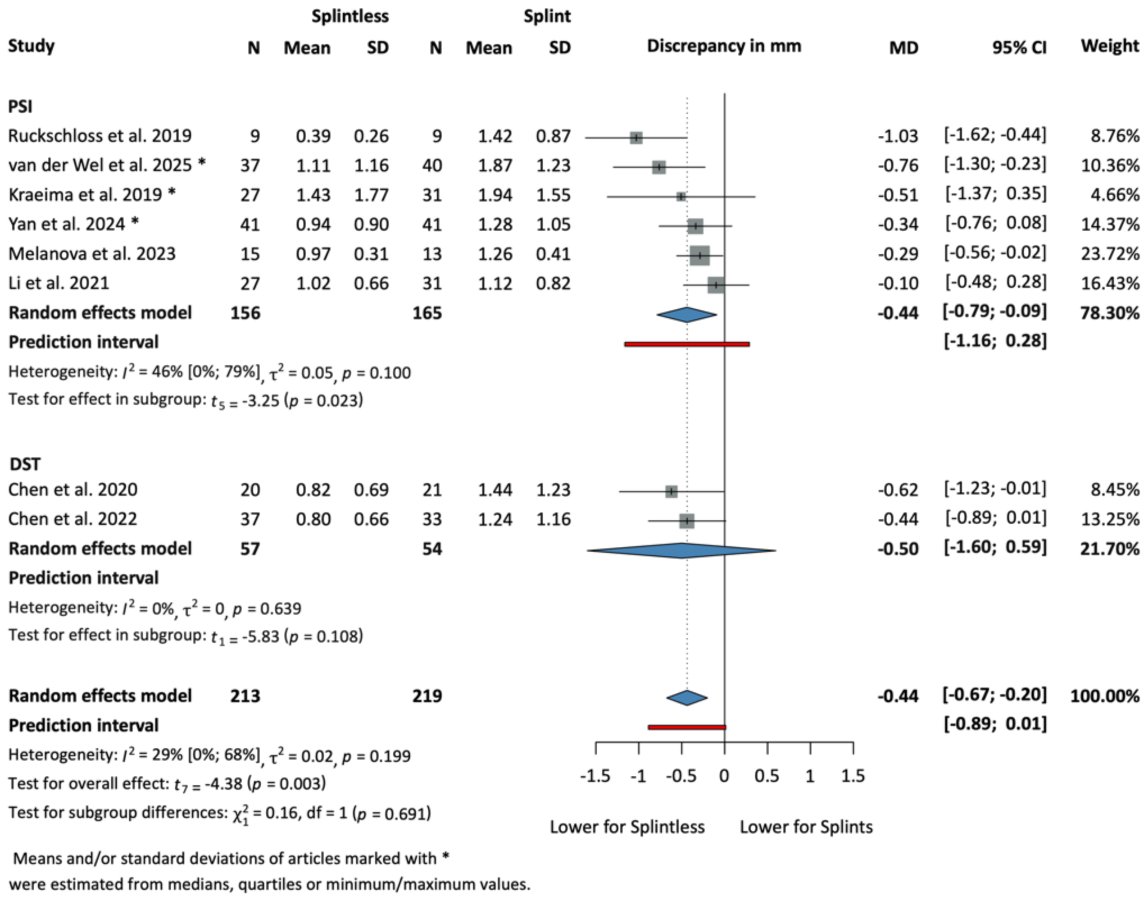

N: number of patients in each group, MD: mean difference, SD: standard deviation, CI: confidence interval, PSI: patient-specific implants, DST: digital surgical templates.

SDC, Figure S2. Forest plot (excluding the study by Liu et al.[7]) demonstrating the effect of splintless (PSI/DST) versus splint-based approaches in LFI osteotomies on the mean deviation from the planned position (in mm) in the mediolateral axis.

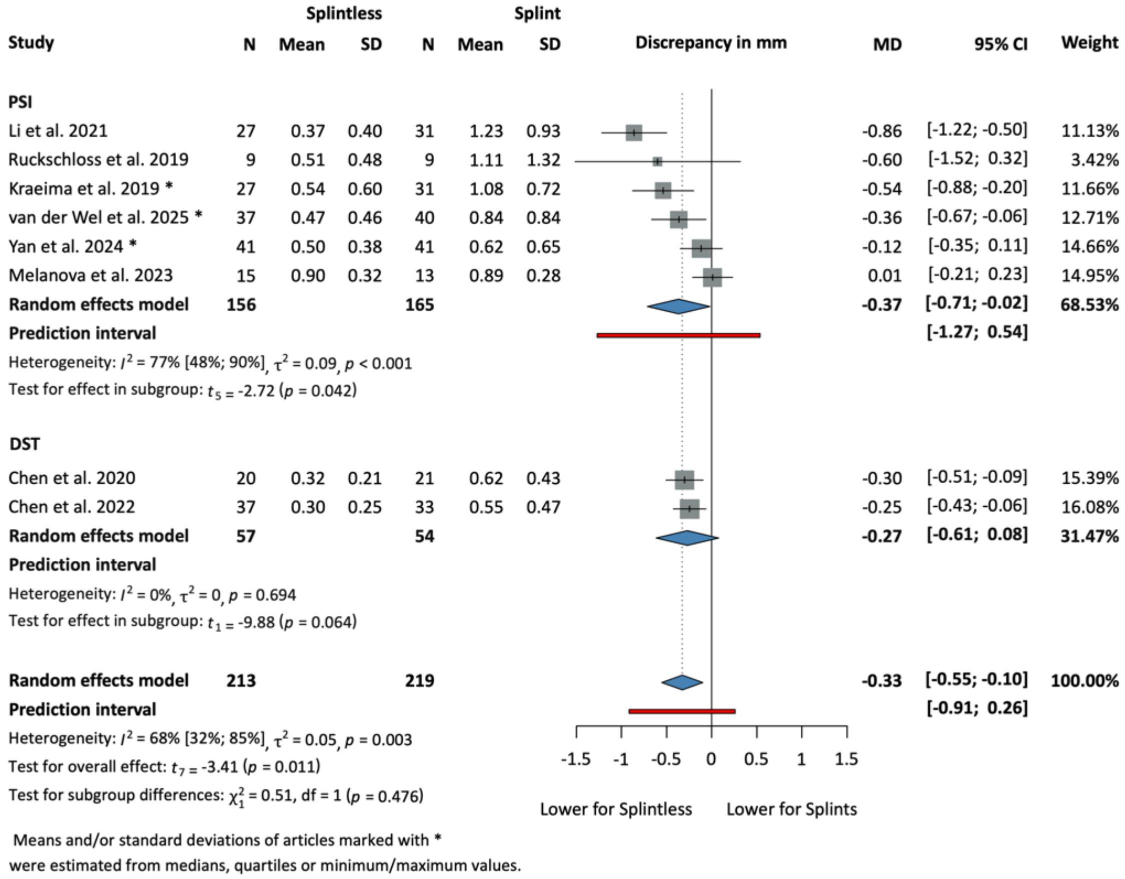

N: number of patients in each group, MD: mean difference, SD: standard deviation, CI: confidence interval, PSI: patient-specific implants, DST: digital surgical templates.

SDC, Figure S3. Forest plot (excluding the study by Liu et al.[7]) demonstrating the effect of splintless (PSI/DST) versus splint-based approaches in LFI osteotomies on the mean deviation from the planned position (in mm) in the vertical axis.

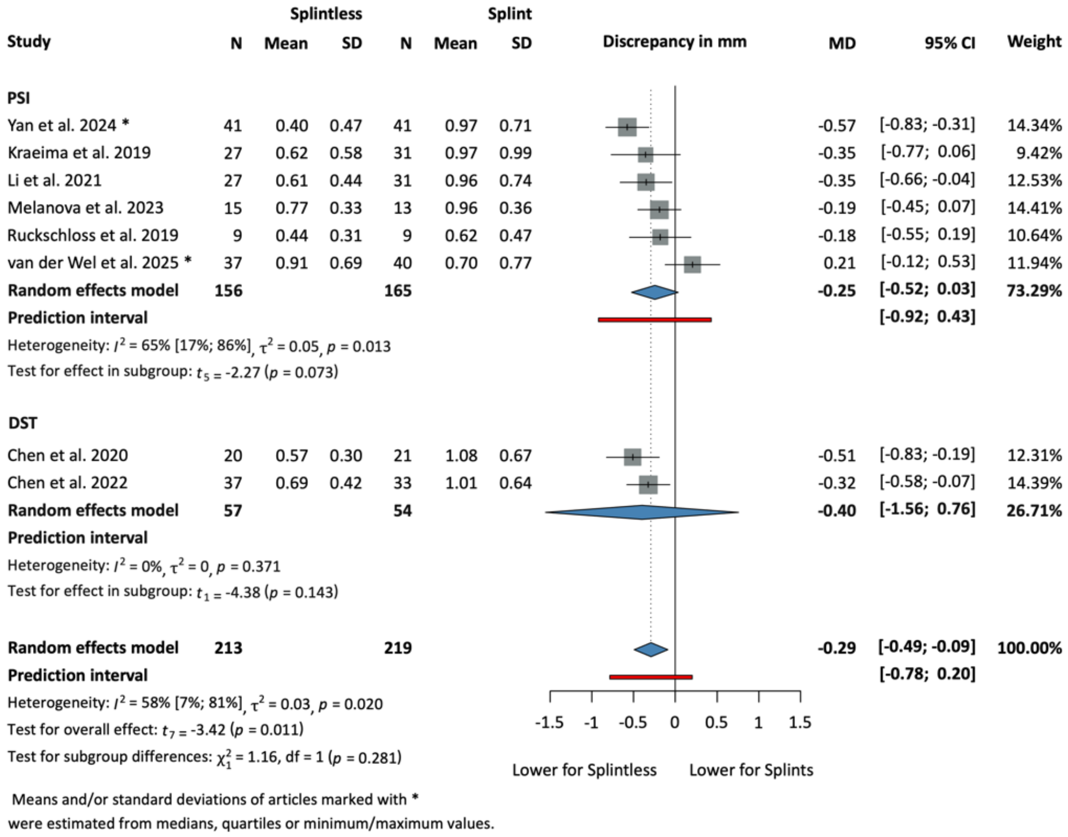

N: number of patients in each group, MD: mean difference, SD: standard deviation, CI: confidence interval, PSI: patient-specific implants, DST: digital surgical templates.

SDC, Figure S4. Forest plot (excluding the study by Liu et al.[7]) demonstrating the effect of PSI versus splint-based approaches in LFI osteotomies on the mean deviation from the planned position (in degrees) in roll.

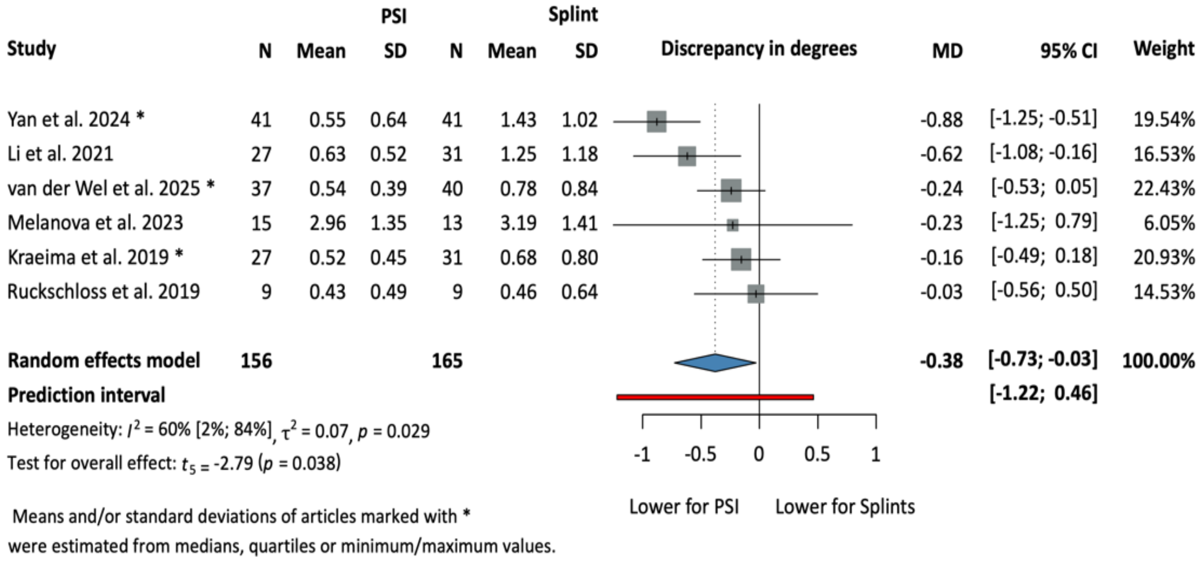

N: number of patients in each group, MD: mean difference, SD: standard deviation, CI: confidence interval, PSI: patient-specific implants.

SDC, Figure S5. Forest plot (excluding the study by Liu et al.[7]) demonstrating the effect of PSI versus splint-based approaches in LFI osteotomies on the mean deviation from the planned position (in degrees) in pitch.

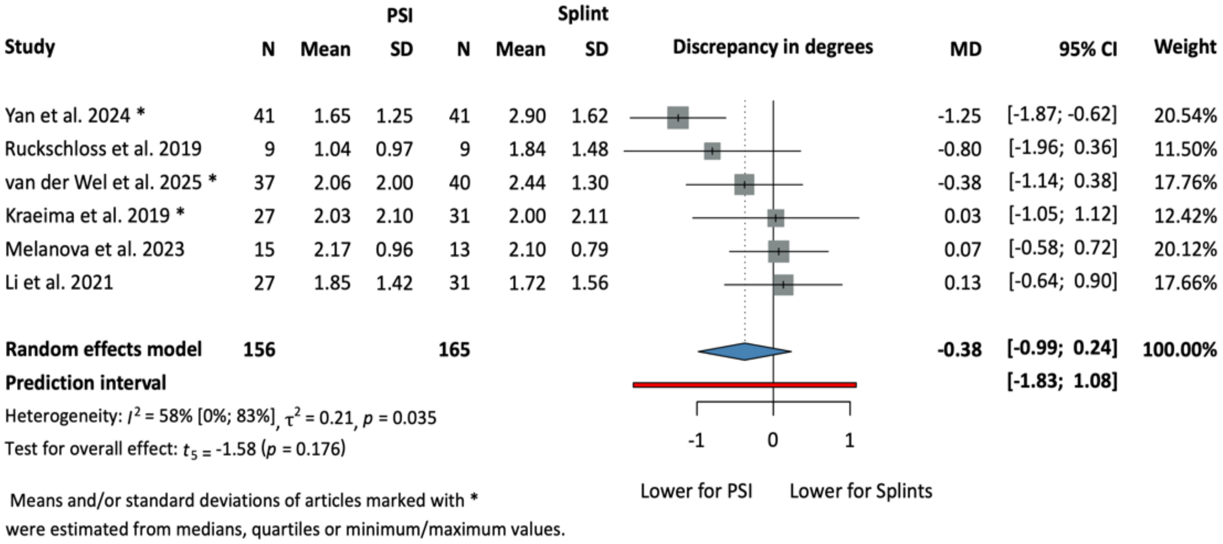

N: number of patients in each group, MD: mean difference, SD: standard deviation, CI: confidence interval, PSI: patient-specific implants.

SDC, Figure S6. Forest plot (excluding the study by Liu et al.[7]) demonstrating the effect of PSI versus splint-based approaches in LFI osteotomies on the mean deviation from the planned position (in degrees) in yaw.

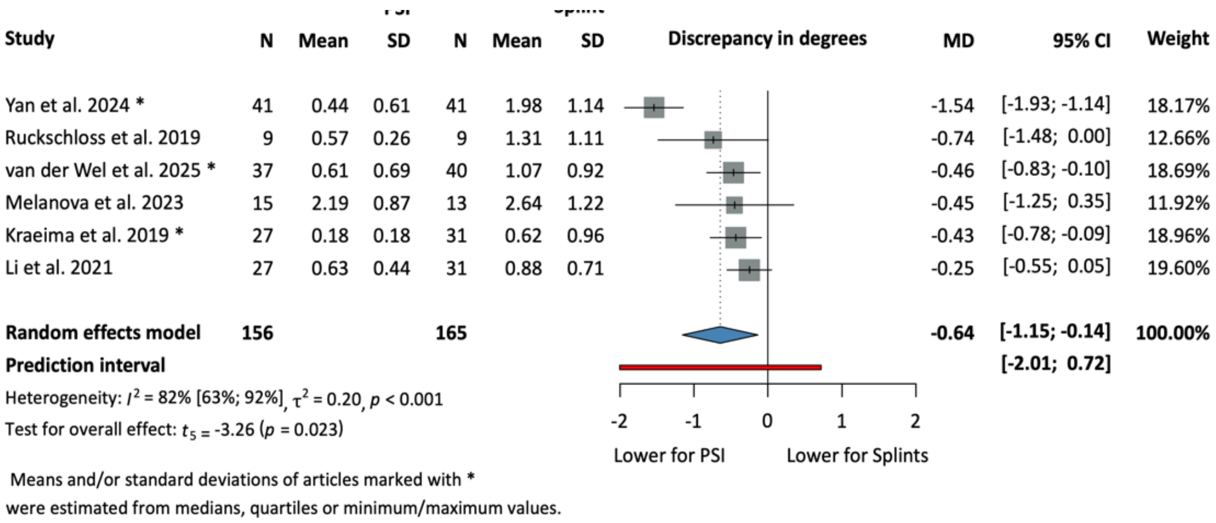

N: number of patients in each group, MD: mean difference, SD: standard deviation, CI: confidence interval, PSI: patient-specific implants.

SDC, Figure S7. Funnel plot assessing the publication bias for the mean deviation from the planned position in the anteroposterior axis.

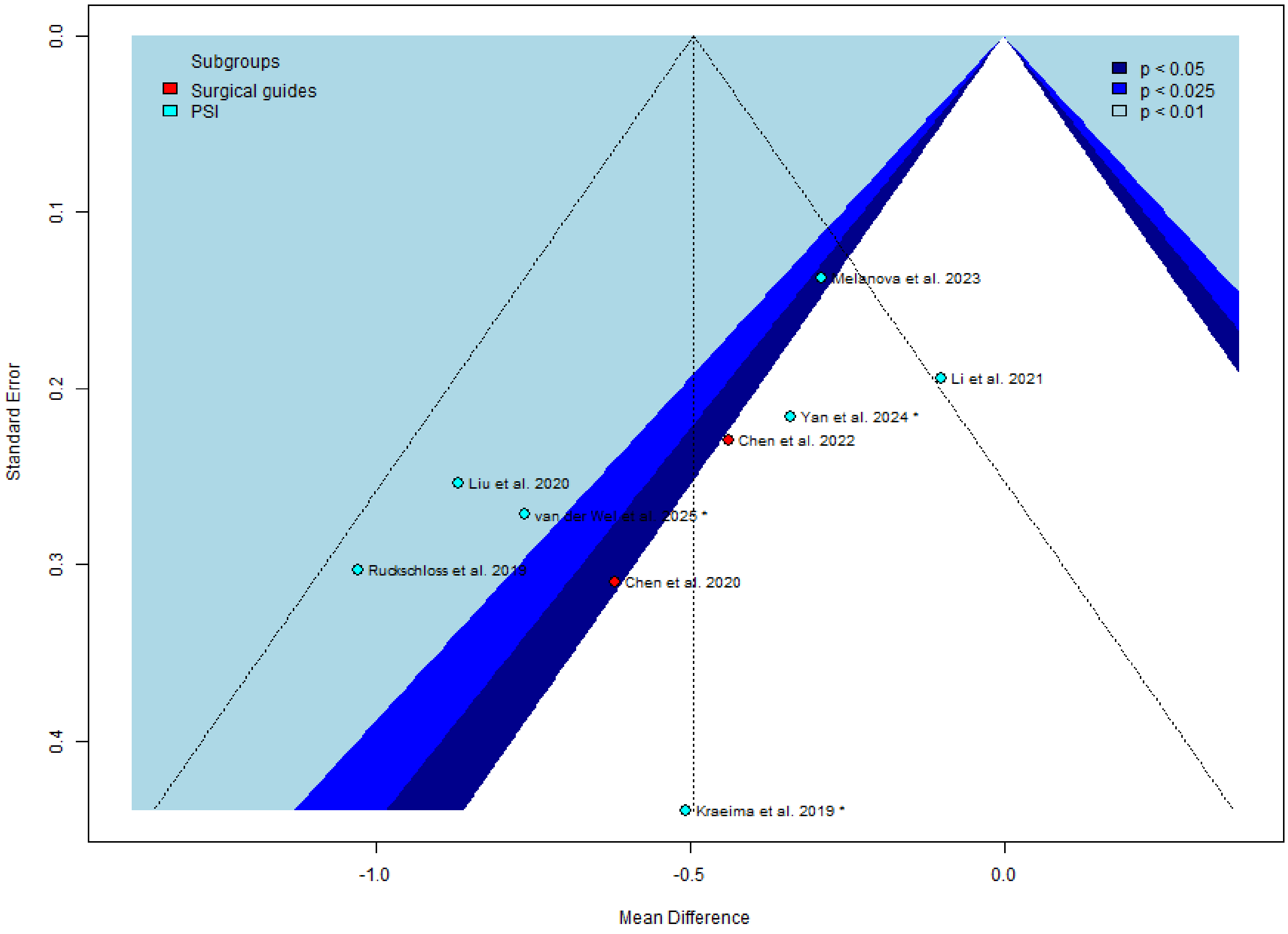

PSI- patient-specific implants

SDC, Figure S8. Funnel plot assessing the publication bias for the mean deviation from the planned position in the mediolateral axis.

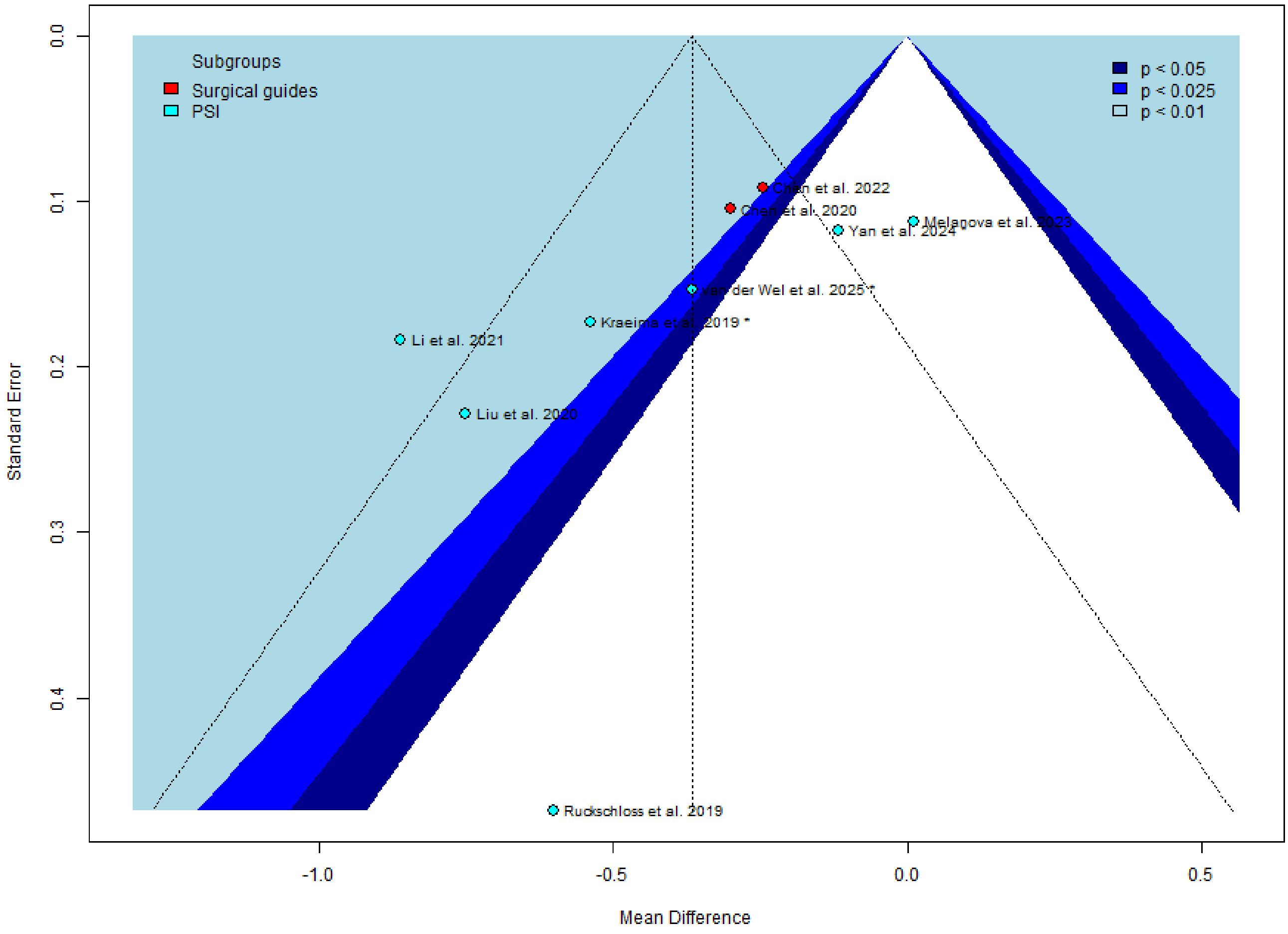

PSI- patient-specific implants

SDC, Figure S9. Funnel plot assessing the publication bias for the mean deviation from the planned position in the vertical axis.

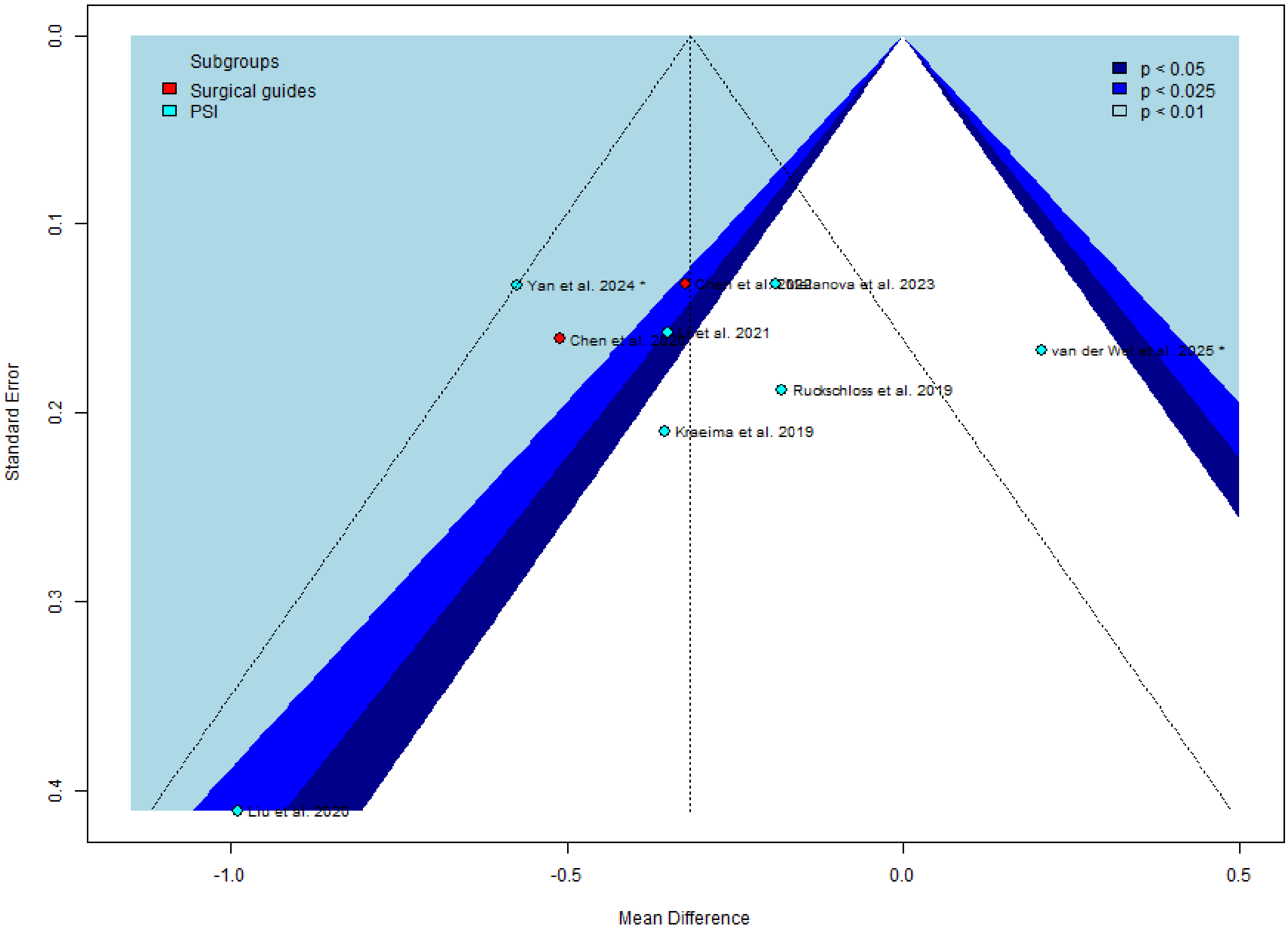

PSI- patient-specific implants

SDC, Figure S10. Funnel plot assessing the publication bias for the mean deviation from the planned position in roll.

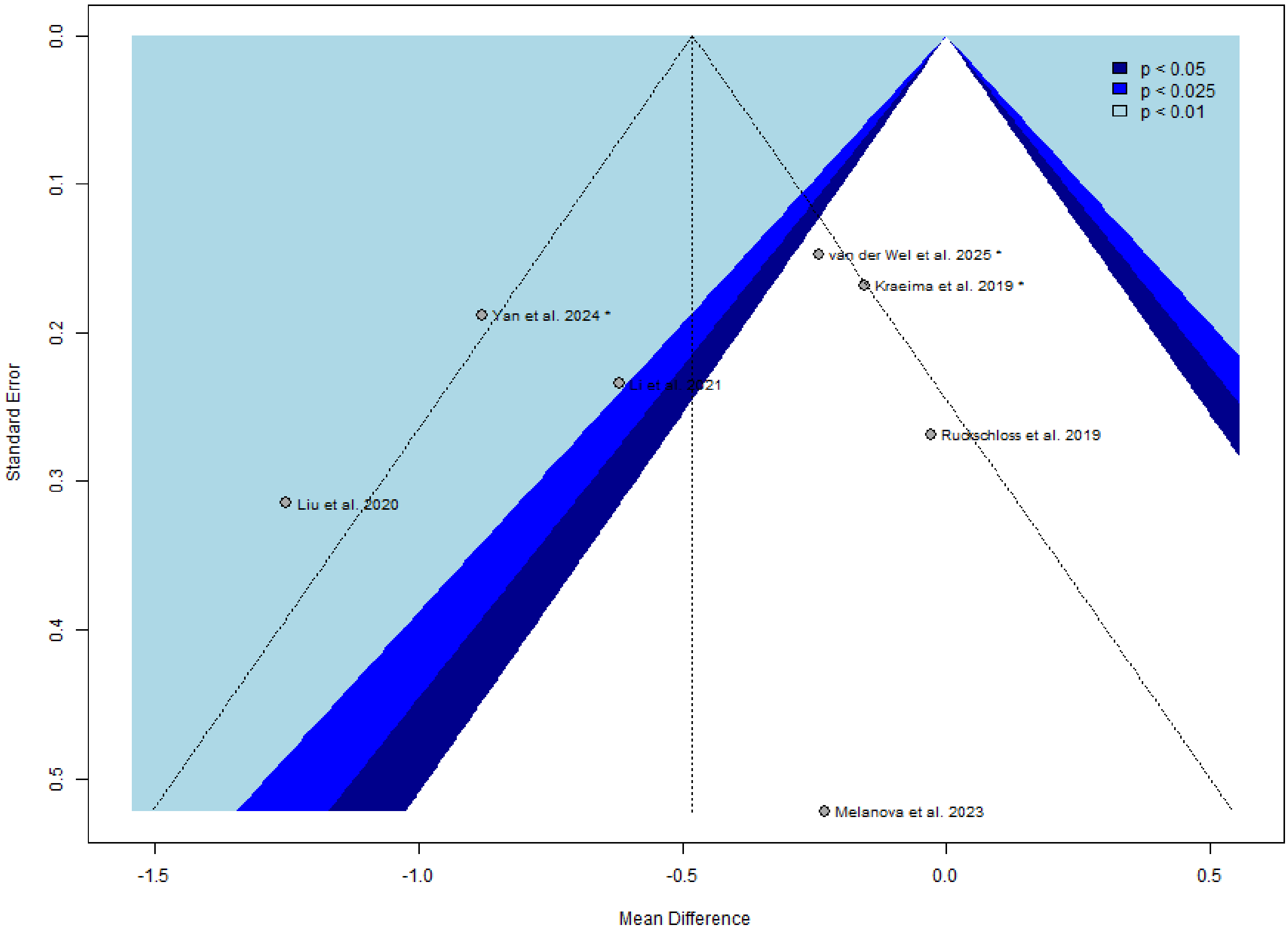

SDC, Figure S11. Funnel plot assessing the publication bias for the mean deviation from the planned position in pitch.

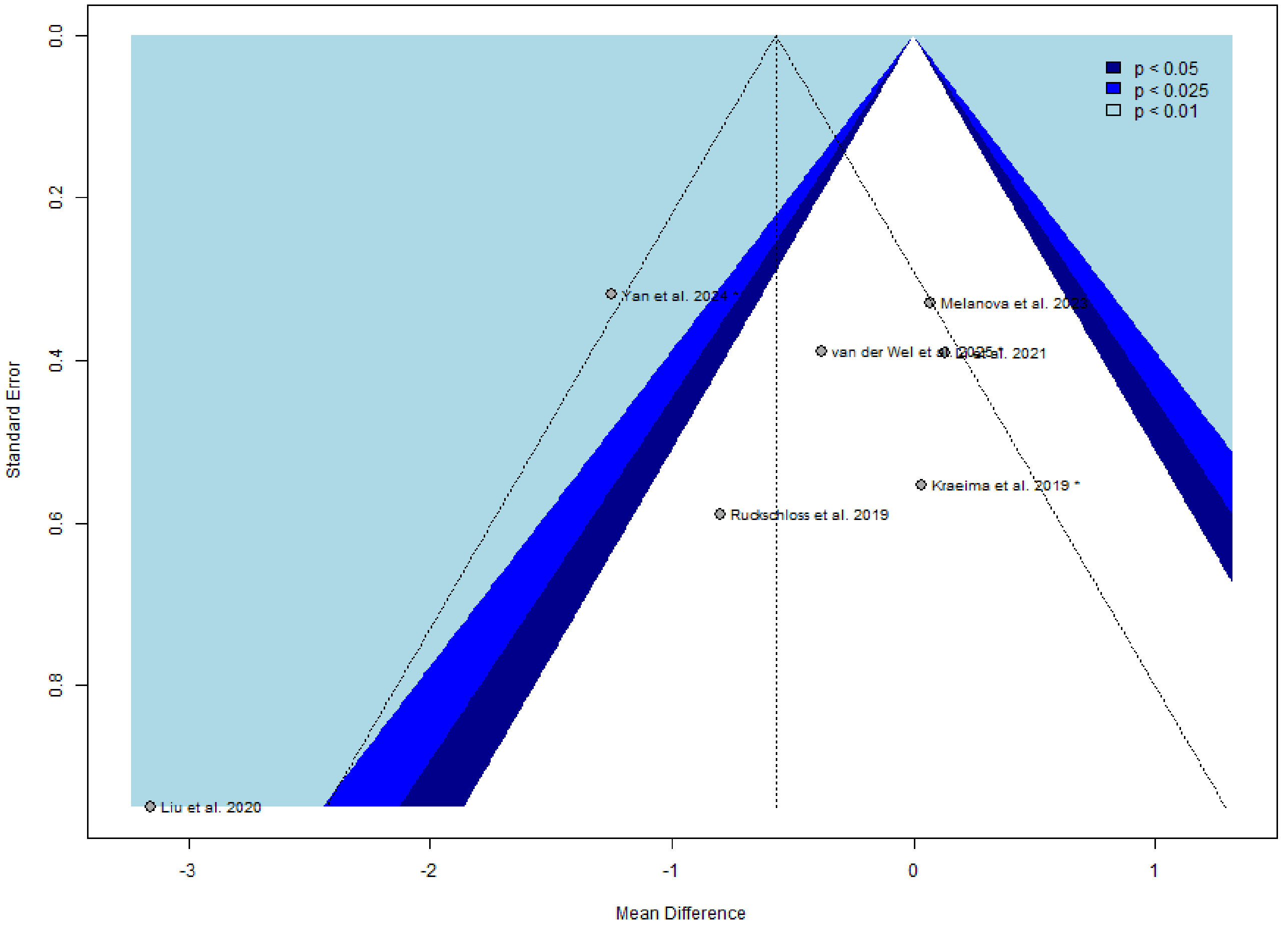

SDC, Figure S12. Funnel plot assessing the publication bias for the mean deviation from the planned position in yaw.

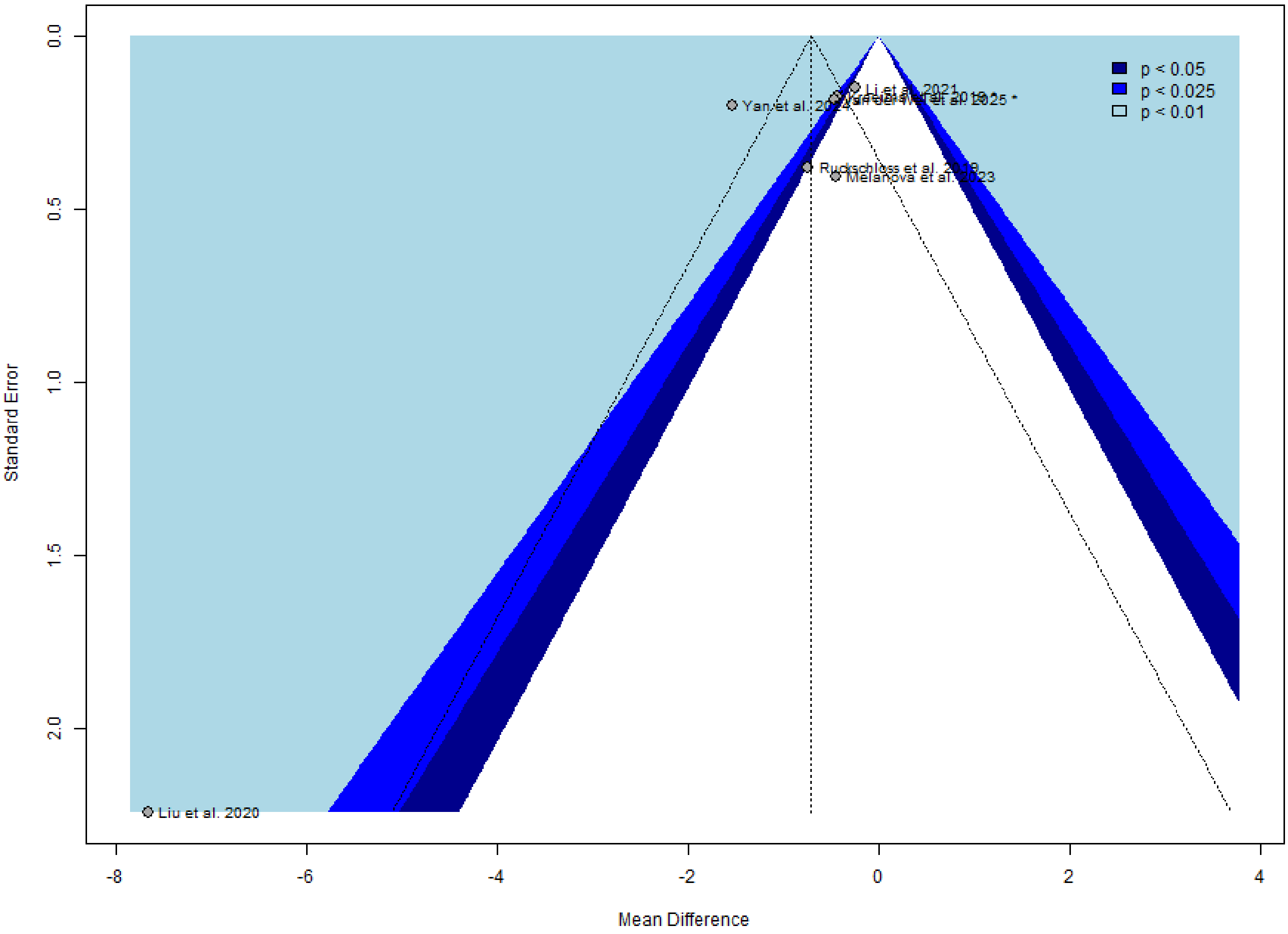

References

1. van der Wel, H., R.H. Schepers, F. Baan, F.K.L. Spijkervet, J. Jansma and J. Kraeima, *Maxilla-first patient-specific osteosynthesis vs mandible-first bimaxillary orthognathic surgery using splints: a randomized controlled trial.* International Journal of Oral and Maxillofacial Surgery, 2025.
2. Yan, J., B. Li, C. Zhang, Y. Qian, Z. Li and X. Wang, *Accuracy of patient-specific implants versus CAD/CAM splints with the mandible-first approach in bimaxillary orthognathic surgery for skeletal Class II malocclusion.* International Journal of Oral and Maxillofacial Surgery, 2024.
3. Malenova, Y., F. Ortner, P. Liokatis, S. Haidari, M. Tröltzsch, F. Fegg, K.T. Obermeier, J.T. Hartung, T.K. Kakoschke, E. Burian, S. Otto, H. Sabbagh and F.A. Probst, *Accuracy of maxillary positioning using computer-designed and manufactured occlusal splints or patient-specific implants in orthognathic surgery.* Clinical oral investigations, 2023. **27**(9): p. 5063-5072.
4. Chen, H., N. Jiang, R. Bi, Y. Liu, Y. Li, W. Zhao and S. Zhu, *Comparison of the Accuracy of Maxillary Repositioning Between Using Splints and Templates in 2-Jaw Orthognathic Surgery.* Journal of oral and maxillofacial surgery, 2022. **80**(8): p. 1331-1339.
5. Chen, H., R. Bi, Z. Hu, J. Chen, N. Jiang, G. Wu, Y. Li, E. Luo and S. Zhu, *Comparison of three different types of splints and templates for maxilla repositioning in bimaxillary orthognathic surgery: a randomized controlled trial.* International journal of oral and maxillofacial surgery, 2021. **50**(5): p. 635-642.
6. Li, B., H. Wei, T. Jiang, Y. Qian, T. Zhang, H. Yu, L. Zhang and X. Wang, *Randomized Clinical Trial of the Accuracy of Patient-Specific Implants versus CAD/CAM Splints in Orthognathic Surgery.* Plastic and reconstructive surgery, 2021. **148**(5): p. 1101-1110.
7. Liu, K., H. Sun, L. Zhang, B. Li, S. Chakraborty and X. Wang, *Do patient-specific cutting guides and plates improve the accuracy of maxillary repositioning in hemifacial microsomia?* British Journal of Oral and Maxillofacial Surgery, 2020. **58**(5): p. 590-596.
8. Kraeima, J., R.H. Schepers, F.K.L. Spijkervet, T.J.J. Maal, F. Baan, M.J.H. Witjes and J. Jansma, *Splintless surgery using patient-specific osteosynthesis in Le Fort I osteotomies: a randomized controlled multi-centre trial.* International journal of oral and maxillofacial surgery, 2020. **49**(4): p. 454-460.
9. Rückschloß, T., O. Ristow, M. Müller, R. Kühle, S. Zingler, M. Engel, J. Hoffmann and C. Freudlsperger, *Accuracy of patient-specific implants and additive-manufactured surgical splints in orthognathic surgery — A three-dimensional retrospective study.* Journal of Cranio-Maxillofacial Surgery, 2019. **47**(6): p. 847-853.
10. Varidel A, Padwa BL, Britt MC, Flanagan S, Green MA. Patient-specific Le Fort I Osteotomy Plates are More Stable than Stock Plates in Patients with Cleft Lip and Palate. *Plast Reconstr Surg*. 2024.
11. Cho JY, Ryu J, Jung S, Kook MS, Park HJ, Oh HK. In-house CAD/CAM fabricated repositioning guide in maxillary repositioning after Le Fort I osteotomy. *J Stomatol Oral Maxillofac Surg*. 2024:102102.
12. van der Wel H, Kraeima J, Spijkervet FKL, Schepers RH, Jansma J. Postoperative skeletal stability at the one-year follow-up after splintless Le Fort I osteotomy using

patient-specific osteosynthesis versus conventional osteosynthesis: a randomized controlled trial. *Int J Oral Maxillofac Surg*. 2023;52(6):679-685.

13. Pietzka S, Fink J, Winter K, et al. Dental Root Injuries Caused by Osteosynthesis Screws in Orthognathic Surgery—Comparison of Conventional Osteosynthesis and Osteosynthesis by CAD/CAM Drill Guides and Patient-Specific Implants. *J Pers Med*. 2023;13(5).
14. Harding J, Hartsfield JK, Mian AS, et al. Accuracy of mandibular proximal segment position using virtual surgical planning and custom osteosynthesis plates. *Int J Oral Maxillofac Surg*. 2022;51(2):219-225.
15. Fleury CM, Sayyed AA, Baker SB. Custom Plates in Orthognathic Surgery: A Single Surgeon's Experience and Learning Curve. *J Craniofac Surg*. 2022;33(7):1976-1981.
16. Cassoni A, Manganiello L, Barbera G, et al. Three-Dimensional Comparison of the Maxillary Surfaces through ICP-Type Algorithm: Accuracy Evaluation of CAD/CAM Technologies in Orthognathic Surgery. *Int J Environ Res Public Health*. 2022;19(18)
